# Mitochondrial oxidative capacity and NAD^+^ biosynthesis are reduced in human sarcopenia across ethnicities

**DOI:** 10.1038/s41467-019-13694-1

**Published:** 2019-12-20

**Authors:** Eugenia Migliavacca, Stacey K. H. Tay, Harnish P. Patel, Tanja Sonntag, Gabriele Civiletto, Craig McFarlane, Terence Forrester, Sheila J. Barton, Melvin K. Leow, Elie Antoun, Aline Charpagne, Yap Seng Chong, Patrick Descombes, Lei Feng, Patrice Francis-Emmanuel, Emma S. Garratt, Maria Pilar Giner, Curtis O. Green, Sonia Karaz, Narasimhan Kothandaraman, Julien Marquis, Sylviane Metairon, Sofia Moco, Gail Nelson, Sherry Ngo, Tony Pleasants, Frederic Raymond, Avan A. Sayer, Chu Ming Sim, Jo Slater-Jefferies, Holly E. Syddall, Pei Fang Tan, Philip Titcombe, Candida Vaz, Leo D. Westbury, Gerard Wong, Wu Yonghui, Cyrus Cooper, Allan Sheppard, Keith M. Godfrey, Karen A. Lillycrop, Neerja Karnani, Jerome N. Feige

**Affiliations:** 1 Nestle Research, EPFL Innovation Park, Lausanne, Switzerland; 2 KTP-National University Children’s Medical Institute, National University Hospital, Singapore, Singapore; 3 Department of Paediatrics, Yong Loo Lin School of Medicine, National University of Singapore, Singapore, Singapore; 4 Medical Research Council Lifecourse Epidemiology Unit, University of Southampton, Southampton, UK; 5 National Institute for Health Research Southampton Biomedical Research Centre, University of Southampton and University Hospital Southampton NHS Foundation Trust, Southampton, UK; 6 Academic Geriatric Medicine, , University of Southampton, Southampton, UK; 7 EPFL school of Life Sciences, Ecole Polytechnique Federale de Lausanne, Lausanne, Switzerland; 8 Department of Molecular & Cell Biology, College of Public Health, Medical & Veterinary Sciences, James Cook University, Townsville, Queensland Australia; 9 UWI Solutions for Developing Countries, UWI SODECO, University of West Indies, Kingston, Jamaica; 10 Singapore Institute for Clinical Sciences (A*STAR), Singapore, Singapore; 11 Department of Endocrinology, Tan Tock Seng Hospital, Singapore, Singapore; 12 Lee Kong Chian School of Medicine, Nanyang Technological University, Singapore, Singapore; 13 Institute of Developmental Sciences, University of Southampton, Southampton, UK; 14 Centre for Biological Sciences, University of Southampton, Southampton, UK; 15 Department of Obstetrics and Gynaecology, Yong Loo Lin School of Medicine, National University of Singapore, Singapore, Singapore; 16 Department of Psychological Medicine, Yong Loo Lin School of Medicine, National University of Singapore, Singapore, Singapore; 17 Liggins Institute, University of Auckland, Auckland, New Zealand; 18 AGE Research Group, Institute of Neuroscience, Faculty of Medical Sciences, Newcastle University, Newcastle, UK; 19 NIHR Newcastle Biomedical Research Centre, Newcastle upon-Tyne NHS Foundation Trust and Newcastle University, Newcastle, UK; 20 National Institute for Health Research Musculoskeletal Biomedical Research Unit, University of Oxford, Oxford, UK; 21 Department of Biochemistry, Yong Loo Lin School of Medicine, National University of Singapore, Singapore, Singapore

**Keywords:** Ageing, Energy metabolism, Skeletal muscle

## Abstract

The causes of impaired skeletal muscle mass and strength during aging are well-studied in healthy populations. Less is known on pathological age-related muscle wasting and weakness termed sarcopenia, which directly impacts physical autonomy and survival. Here, we compare genome-wide transcriptional changes of sarcopenia versus age-matched controls in muscle biopsies from 119 older men from Singapore, Hertfordshire UK and Jamaica. Individuals with sarcopenia reproducibly demonstrate a prominent transcriptional signature of mitochondrial bioenergetic dysfunction in skeletal muscle, with low PGC-1α/ERRα signalling, and downregulation of oxidative phosphorylation and mitochondrial proteostasis genes. These changes translate functionally into fewer mitochondria, reduced mitochondrial respiratory complex expression and activity, and low NAD^+^ levels through perturbed NAD^+^ biosynthesis and salvage in sarcopenic muscle. We provide an integrated molecular profile of human sarcopenia across ethnicities, demonstrating a fundamental role of altered mitochondrial metabolism in the pathological loss of skeletal muscle mass and function in older people.

## Abstract (web summary)

Sarcopenia is the loss of muscle mass and strength associated with physical disability during ageing. Here, the authors analyse muscle biopsies from 119 patients with sarcopenia and age-matched controls of different ethnic groups and find transcriptional signatures indicating mitochondrial dysfunction, associated with reduced mitochondria numbers and lower NAD^+^ levels in older individuals with sarcopenia.

## Introduction

Loss of muscle mass and strength is a fundamental feature of aging. Both muscle mass and muscle strength decline between 3 and 8% per decade after midlife, with rates accelerating after age 60 years^1^. Age-related muscle decline is driven by lifestyle, endocrine, nutritional, and cellular causes that are well-studied in healthy older populations^2,3^. Preclinical and human studies comparing young and aged individuals suggest that chronic low-grade inflammation^4^, loss of anabolic signaling through GH/IGF-1 (ref. ^5^), lower protein intake and vitamin D insufficiency^6^ contribute to a loss of muscle plasticity during aging. Systemic signals cross-talk with intrinsic mechanisms, which compromise muscle quality at the cellular level through impaired anabolic signaling which reduces protein synthesis, increased myosteatosis, cycles of myofiber denervation and innervation, altered cellular quality control by autophagy, loss of regenerative potential through stem cell dysfunction, and perturbed bioenergetics^7–9^. Efficient skeletal muscle bioenergetics largely relies on metabolic flexibility in mitochondria and robust mito-hormesis to integrate mitochondrial function with the rest of the cell and the organism^10,11^. Mitochondrial function in skeletal muscle declines during aging, concomitant with a decrease in exercise and physical activity^2,12^. Aged muscle fibers have an impaired capacity to oxidize metabolic fuels in mitochondria, and previous studies have implicated reduced skeletal muscle mitochondrial biogenesis, expression of mitochondrial respiratory complex subunits, mitochondrial respiration, and ATP levels^13,14^. Excessive free radical production by the electron transfer chain and altered reactive oxygen species detoxification have been suggested to cause cumulative damage during aging, leading to the impairment of mitochondrial function through mitochondrial protein oxidation and mutations within mtDNA^15^. In addition, work in model organisms has demonstrated that aging impairs mitochondrial dynamics^16^, and the ability to repair or recycle damaged mitochondria through the mitochondrial unfolded protein response (UPRmt) and mitophagy^17,18^.

While the molecular processes associated with the average functional decline of muscle in the healthy population have been well studied, little is known about the pathological state of sarcopenia, the pathological muscle wasting and weakness of the old age recently assigned an ICD-10 disease code due to its negative impact on physical function, quality of life, and survival^6,19–21^. Sarcopenia is defined clinically by low muscle mass and functional impairments of mobility and muscle strength, using population cutoffs which define the subset of the older population with the highest risk of physical dysfunction^22,23^. Sarcopenia predicts future disability and mortality^19,24^, and associates with high healthcare costs^25^. Prevalence estimates of sarcopenia vary according to the age group, operational definition used and clinical setting, ranging from 2 to 20% in community dwelling older people and up to 33% among patients in long term care^26^. Recognized influences contributing to the variability between older individuals include age, gender, developmental plasticity, fixed genetic factors, physical activity, nutrition, and co-morbidities^6,19,27,28^, but much variation remains unexplained. Standardization of the clinical definition now provides an opportunity to characterize the molecular “signature” of sarcopenia in older people.

Here, we report a novel multi-ethnic study comparing the genome-wide transcriptomic profiles of skeletal muscle biopsies from older men diagnosed with sarcopenia with age-matched controls using high coverage RNA sequencing (MEMOSA—Multi-Ethnic Molecular determinants of Sarcopenia). For the first time we demonstrate that mitochondrial bioenergetic dysfunction is the strongest molecular signature of sarcopenia in three distinct ethnic populations, with major impairments of oxidative phosphorylation, mitochondrial dynamics, and mitochondrial quality control through the UPRmt. Mechanistically, this associates with the downregulation of an ERRα/PGC-1α/NRF1 regulatory module, lower NAD^+^ levels and alterations of mitochondrial respiratory complex protein expression and activity in human sarcopenia.

## Results

Twenty community-dwelling sarcopenic men of Chinese descent and 20 age-matched controls were recruited in Singapore (Singapore sarcopenia study, SSS, mean age 71.5 years); SSS findings were validated using existing cohorts in the UK (Hertfordshire sarcopenia study, HSS) and Jamaica (Jamaica sarcopenia study, JSS) (Supplementary Table 1). Sarcopenia was defined based on harmonized consensus clinical definitions of the AWGSOP (SSS) or EWGSOP (HSS/JSS)^26^, using skeletal muscle mass evaluation by DXA measurement of appendicular lean body mass index (ALMi), grip strength, and gait speed (Supplementary Table 2). Genome-wide transcriptome was profiled on vastus lateralis muscle biopsies using high coverage total RNA sequencing with >70 million reads per sample (Supplementary Data 1).

### Mitochondrial dysfunction is the major transcriptional hallmark of sarcopenic muscle

**Fig. 1:**
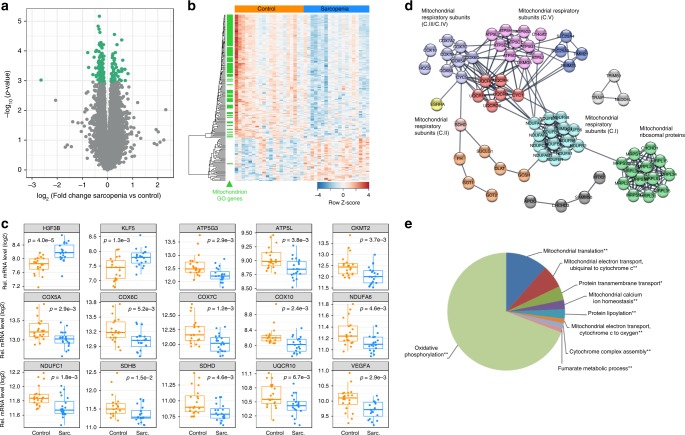
RNA sequencing of human skeletal muscle in SSS. **a** Volcano plot of differentially expressed genes in skeletal muscle of sarcopenic vs. age-matched healthy older people. *p*-values were calculated using moderated t-statistic. The 133 genes downregulated and 46 genes upregulated in sarcopenic muscle using a false-discovery rate (FDR) < 10% are represented in green. **b** Heatmap showing the 179 genes differentially expressed from (**a**) with FDR < 10%. Genes belonging to the cellular component GO term “mitochondrion” (GO:0005739) are labeled with a green tick. **c** Validation of gene expression changes in sarcopenic muscle of SSS for selected genes using quantitative mRNA profiling by nanoString nCounter; mRNA expression values are normalized to ten stable housekeeping genes. *n* = 40 muscle samples analyzed with a two sided *t* test. **d** Network representation of the protein-protein interactions of genes differentially regulated in sarcopenic muscle at FDR < 10% using STRING. Nodes with an interaction score > 0.9 are represented and colored by biological function. **e** Gene ontology enrichment of the genes regulated in sarcopenic muscle. Pie-chart represents the % of differentially expressed genes, *p < 0.05 and **p < 0.01 based on hypergeometric distribution tests. In **a**, **b**, **d**, **e**, *n* = 19–20 muscle samples per group from SSS participants.

**Fig. 2:**
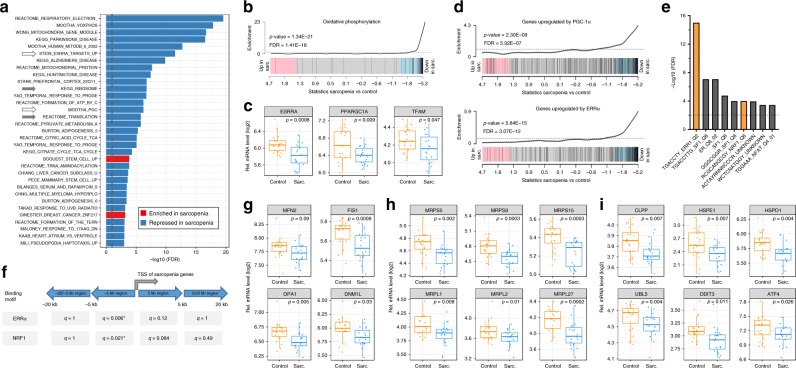
Mitochondrial dysfunction is the major transcriptional change during sarcopenia in SSS. **a** Gene-set enrichment analysis of sarcopenic vs control muscle using CAMERA and the C2 curated gene set collection from MSigDB. Gene sets are ordered according to the significance of their enrichment; only gene sets with an FDR < 0.1% and a gene overlap < 75% are represented. White arrows highlight gene sets linked to the transcriptional regulation of mitochondrial function; gray arrows highlight gene sets linked to protein synthesis. **b** Enrichment plot for the oxidative phosphorylation gene set “Mootha_VOxPhos; M18264”. **c** mRNA expression of transcriptional regulators of mitochondrial function in SSS sarcopenic vs. control muscle. **d** Enrichment plot for PGC-1α target genes and ERRα target gene set (“Mootha_PGC1a; M9788” and “Stein_ESRRa_Up; M18491”). **e** Transcription factor binding site enrichment of the 4 kb promoters of genes regulated in sarcopenic muscle at FDR *q*-value < 0.05. *x*-axis represents the MsigDB transcription factor gene sets that passed the significance threshold. **f** ERRα and NRF1 binding motif in the proximal and distal regions flanking the transcriptional start site (TSS) of the genes regulated in sarcopenic muscle at FDR *q*-value < 0.05. Benjamini Hochberg-corrected median-adjusted *q*-values were computed by performing 1000 hypergeometric test permutations. **g**–**i** mRNA expression of genes regulating mitochondrial dynamics (**g**), mitochondrial ribosomal protein genes (**h**), and UPRmt genes (**i**) in SSS sarcopenic vs. control muscle. In **c**, **g**–**i**, nominal *p* values of the moderated t-statistic are reported. For all panels, *n* = 19–20 muscle samples per group from SSS participants.

Case–control analysis in SSS revealed a strong perturbation of muscle gene expression in sarcopenic participants where 179 genes encoding 150 proteins and 29 noncoding RNAs were altered in sarcopenic muscle with false-discovery rate (FDR) < 10% (Fig. 1a; Supplementary Data 2). This sarcopenic signature was enriched in downregulated genes, out of which 133 genes were annotated under the mitochondrion gene ontology term (Fig. 1b, green ticks). Independent mRNA gene expression validation of 80 selected genes using nanoString nCounter demonstrated tight correlation with RNAseq data (Supplementary Fig. 1a; Supplementary Data 3), and confirmed lower expression of mitochondrial function genes in sarcopenic muscle (Fig. 1c). Network and gene ontology analysis of downregulated genes distinguished several clusters linked to mitochondrial respiratory chain complexes, oxidative phosphorylation and mitochondrial translation (Fig. 1d, e). Mitochondrial alterations were also confirmed as the strongest signature in sarcopenic muscle in pathway enrichment analyses using CAMERA (Fig. 2a, b; Supplementary Data 4). Mitochondrial respiratory chain, TCA cycle regulator, and oxidative phosphorylation gene sets were repressed in sarcopenic muscle with highly significant FDRs (<10E−10). Gene sets affected by age-related neurodegenerative diseases such as Alzheimer disease were also downregulated in sarcopenic muscle (Fig. 2a), but the enrichment of these gene sets was caused by regulators of mitochondrial function (Supplementary Fig. 1b) that are also altered during neurodegeneration^29^. Mitochondrial function in skeletal muscle declines throughout the life course through impaired mitochondrial biogenesis, expression of mitochondrial respiratory complex subunits, mitochondrial respiration, and ATP levels during aging^2,12^. To uncouple the pathological drivers of sarcopenia from the general effect of aging on mitochondrial function, we adjusted the genome-wide RNAseq analyses for age. Age-adjustment slightly decreased the statistical significance of the differentially expressed genes (Supplementary Fig. 1c), but the rank of genes differentially expressed in sarcopenic muscle was highly conserved (Supplementary Fig. 1d, Spearman rank correlation *r*_s_ = 0.95). Mitochondrial function and oxidative phosphorylation remained the strongest downregulated hallmarks of sarcopenic muscle in age-adjusted pathway enrichment analyses (Supplementary Fig. 1e). Thus, mitochondrial energy production is the strongest transcriptional signature of sarcopenia and pathological muscle dysfunction.

### Altered signaling through ERRα and PGC-1α in sarcopenic muscle

The pathway enrichment analyses of sarcopenic muscle also revealed lower expression of the transcriptional networks regulated by the ERRα nuclear receptor (gene name *ESRRA*) and the PGC-1α transcriptional coactivator (*PPARGCA1*) (Fig. 2a), while the mRNAs of the energy sensor AMP-activated kinase (AMPK) and its downstream targets were not changed during sarcopenia (Supplementary Fig. 2a–c). mRNA levels of *PGC-1α* and *ERRα* were reduced in sarcopenic muscle (Fig. 2c), and downstream targets including *TFAM*^30^ were also downregulated (Fig. 2c, d). In addition, the promoters of the genes downregulated in sarcopenic muscle were highly enriched in *ERRα* and nuclear respiratory factor 1 (*NRF1)* binding sites (Fig. 2e, f). The transcriptional regulators ERRα and NRF1 and their coactivator PGC-1α have been widely demonstrated to regulate mitochondrial gene expression in rodents and humans^30,31^. In particular, overexpression of PGC-1α or ERRα is sufficient to induce the expression of genes controlling mitochondrial activity and to trigger functional benefits on oxidative phosphorylation and ATP generation. Thus, reduced transcriptional activity of ERRα and of PGC-1α-dependent transcription factors in sarcopenic muscle may contribute to the global mitochondrial alterations observed in sarcopenia.

### Perturbed mitochondrial dynamics and UPRmt in sarcopenic muscle

Expression profiles of genes controlling mitochondrial dynamics through fusion and fission were lower in sarcopenic individuals, both through single gene and pathway enrichment analyses (Fig. 2g; Fig. S2d). Our protein association network analysis of genes with altered expression in sarcopenic muscle also revealed a particularly striking node containing mitochondrial ribosomal protein (*MRP*) genes (Fig. 1d). Many genes encoding both the small and large subunits of the mitochondrial ribosome were downregulated in sarcopenic muscle (Fig. 2h; Supplementary Fig. 2e), demonstrating that sarcopenia associates with specific deficits of mitochondrial protein synthesis. MRPs are also important to balance mito-nuclear communication during aging and regulate a protective mitochondrial UPRmt important for the regulation of health span and longevity in preclinical models^32^. Interestingly, many genes controlling the UPRmt were strongly downregulated in the muscle of sarcopenic participants, including those encoding the mitochondrial heat-shock proteins, the protease *CLPP* and their transcriptional effectors *UBL5, ATF4*, and *CHOP/DDIT3* (Fig. 2i; Supplementary Fig. 2f). Thus, inefficient UPRmt activation during sarcopenia fails to compensate the lower production of mitochondrial proteins and their damage induced by oxidative stress^10,17^.

### Sarcopenia has a common transcriptional profile across ethnicities

**Fig. 3:**
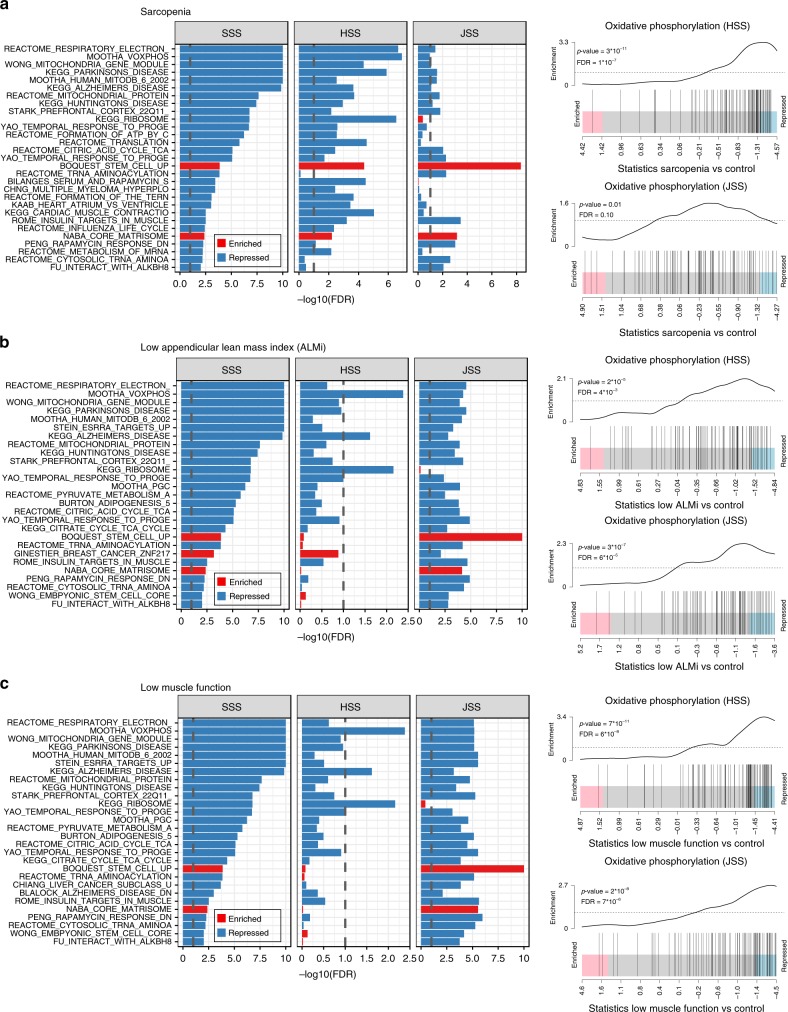
The transcriptional downregulation of mitochondrial bioenergetics in people with sarcopenia and low physical function is replicated in the HSS and JSS cohorts. Gene-set enrichment analysis on muscle RNA expression in the discovery cohort (SSS) and two replication cohorts of different ethnicity (HSS and JSS) using CAMERA and the C2 curated gene set collection from MSigDB. **a** Sarcopenia vs. control in SSS, HSS, and JSS cohorts. **b** Low appendicular lean mass index vs. control in SSS, HSS, and JSS cohorts. **c** Low muscle function (grip strength or gait speed) vs. control in SSS, HSS, and JSS cohorts. In the left panels, gene sets are ordered according to the significance of their association in the SSS cohort; only gene sets with an overlap between sets <75% and an FDR < 1% in SSS and at least one other cohort are reported. The significance threshold of 10% FDR is represented by dashed gray lines and FDRs smaller than 10E−10 are trimmed. Right panels represent the enrichment plots for the “Mootha VOXPHOS” oxidative phosphorylation gene sets in the HSS and JSS cohorts (MSigDB reference M18264). For all panels, *n* = 39 (SSS and JSS) and *n* = 40 (HSS) muscle samples per cohort were stratified in the different phenotypes as described in Supplementary Table 2.

The prevalence of sarcopenia differs by country^26^, but potential differences in etiology across ethnic groups have been little studied. To confirm the prominent role of mitochondrial alterations in human sarcopenia observed in the SSS cohort, we used preexisting cohorts of Caucasian (HSS, UK) and Afro-Caribbean (JSS, Jamaica) men with sarcopenia. Gene-set enrichment analysis following high coverage RNA sequencing of sarcopenic and control muscle confirmed that mitochondrial bioenergetic dysfunction is a strong signature of sarcopenia in all cohorts/ethnicities (Fig. 3a), including lower oxidative phosphorylation, mitochondrial respiratory ETC and TCA cycle at FDR < 10E−04 and 10% in HSS and JSS, respectively (Fig. 3a). Gene sets controlling mitochondrial function were also consistently depleted in participants with low ALMi (Fig. 3b) and low muscle function (Fig. 3c) in HSS and JSS cohorts. Together, transcriptomic analyses in the three independent cohorts confirmed a prominent contribution of genes controlling mitochondrial energy production and oxidative phosphorylation in maintaining muscle mass and function in older individuals of different ethnicity.

### Role of other molecular processes in human sarcopenia

Beyond mitochondria, other signatures contributed to the molecular perturbations of human sarcopenic muscle. In particular, ribosome and translation ontologies were overrepresented in the genes downregulated in sarcopenia (Fig. 2a; Supplementary Fig. 2g). Altered muscle protein synthesis has been widely associated with low muscle mass in older people^33^, through a state of anabolic resistance where anabolic hormones and dietary amino acids fail to efficiently promote mTOR signaling and contractile protein synthesis in myofibers^34^. As a sign of altered protein anabolism, mTOR signaling was repressed in sarcopenic muscle (FDR = 1.2E−02; Supplementary Fig. 2g), but to a much lesser extent than oxidative phosphorylation (FDR < 10E−10; Supplementary Fig. 1b). Myofiber denervation and altered neuromuscular junction (NMJ) morphology have been proposed as cellular causes of muscle dysfunction during aging^8^. Strikingly, none of the transcriptional signatures of denervation detected in rodent models were observed in our study (Supplementary Fig. 2h). Sets of genes controlling neuromuscular processes, NMJ structure and acetylcholine receptor signaling were not deregulated in sarcopenic muscle across the three cohorts (Supplementary Fig. 2i, k, l), suggesting that neuromuscular dysfunction is not a major transcriptional mechanism of human sarcopenia. Chronic low-grade inflammation has also been proposed to contribute to sarcopenia through systemic cytokine changes and local targeted responses in skeletal muscle^35^. However, the transcriptional profiles of sarcopenic muscle did not detect inflammatory responses or perturbed signaling through typical pro-inflammatory signaling pathways like JAK/STAT and NFκB in SSS and HSS (Supplementary Fig. 2j, k). JSS revealed a weak inflammatory response in sarcopenic muscle (Supplementary Fig. 2l), suggesting that inflammation may associate with sarcopenia only in a specific subset of people.

### Muscle mass and strength drive the molecular phenotype of sarcopenia

**Fig. 4:**
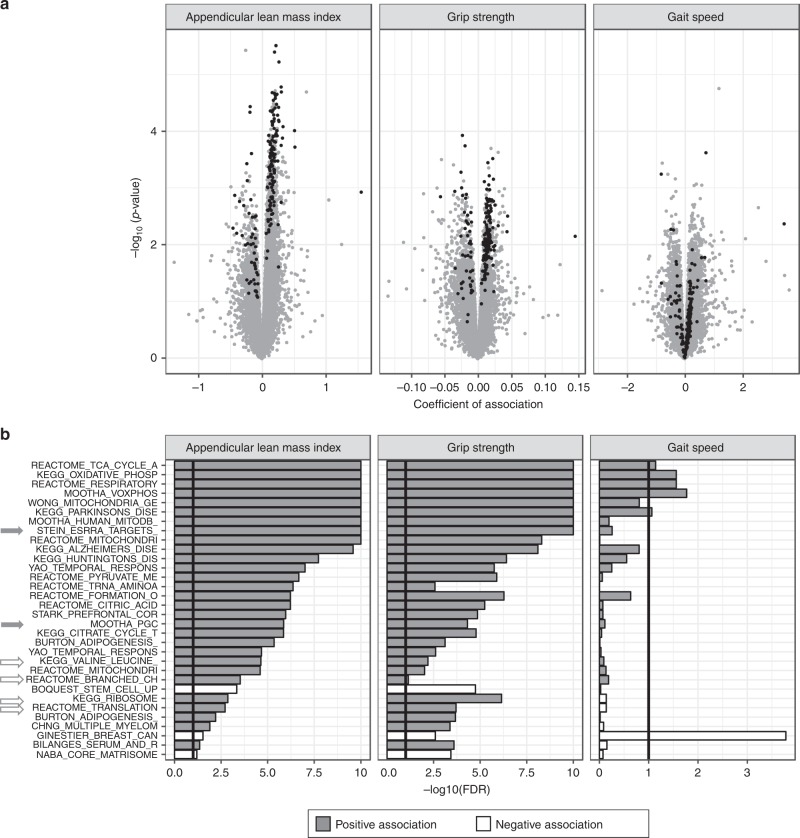
The transcriptional signature of sarcopenia in human muscle is mainly driven by the loss of appendicular lean mass index (ALMi) and grip strength. **a** Volcano plot of genes associated with ALMi, grip strength and gait speed as continuous variables. *p*-values were calculated using moderated *t*-statistics and coefficients of association represent the log2 fold change of gene expression per unit of variable of interest. Genes which are differentially regulated with sarcopenia at a FDR < 10% (Fig. 1a) are represented in black. **b** Gene-set enrichment analysis of genes associated with ALMi, grip strength, and gait speed as continuous variables using CAMERA and the C2 curated gene set collection from MSigDB. Gene sets are ordered according to the significance of their association with ALMi; only gene sets with an FDR < 10% in at least one association and with a gene overlap <75% are represented. The significance threshold of 10% FDR is represented by black vertical lines and FDRs smaller than 10E−10 are trimmed at 10E−10. For all panels, *n* = 39 SSS muscle samples.

To further understand the contribution of muscle mass and function, we performed a genome-wide association of muscle gene expression to ALMi, grip strength and gait speed as continuous variables (Fig. 4a). The expression of 318 genes encoding 276 proteins and 42 noncoding RNAs were associated with ALMi at FDR < 10%. In contrast, muscle gene expression associations with grip strength and gait speed were weaker with only 7 and 9 genes, respectively, associated at a nominal *p* value < 0.001 but FDR > 10%. A large number of genes regulated in sarcopenic muscle were also among the most associated with ALMi and grip strength, but not with gait speed (Fig. 4a, black dots). At the pathway level, mitochondrial function was the strongest biological process associated with muscle mass and muscle function as continuous variables (Fig. 4b). In particular, genes positively correlated with ALMi and grip strength were strongly enriched for TCA cycle, oxidative phosphorylation and mitochondrial respiratory chain gene sets (FDRs < 10E−10). These gene sets related to mitochondrial bioenergetics were also modestly enriched in genes positively associated to gait speed (FDR < 10%). Thus, genes controlling mitochondrial function positively associated with all continuous parameters of sarcopenia. Pathway enrichment analyses also confirmed positive association of ALMi and grip strength with ERRα and PGC-1α transcriptional networks (Fig. 4b, gray arrows), and with gene sets regulating branched-chain amino acid metabolism, ribosomal function and translation (Fig. 4b, white arrows). Together, these results demonstrate that all clinical parameters used to diagnose sarcopenia contribute to the molecular profiles of sarcopenic muscle, with the strongest contribution from low muscle mass followed by grip strength and gait speed.

### Mitochondrial bioenergetics is low in human sarcopenic muscle

**Fig. 5:**
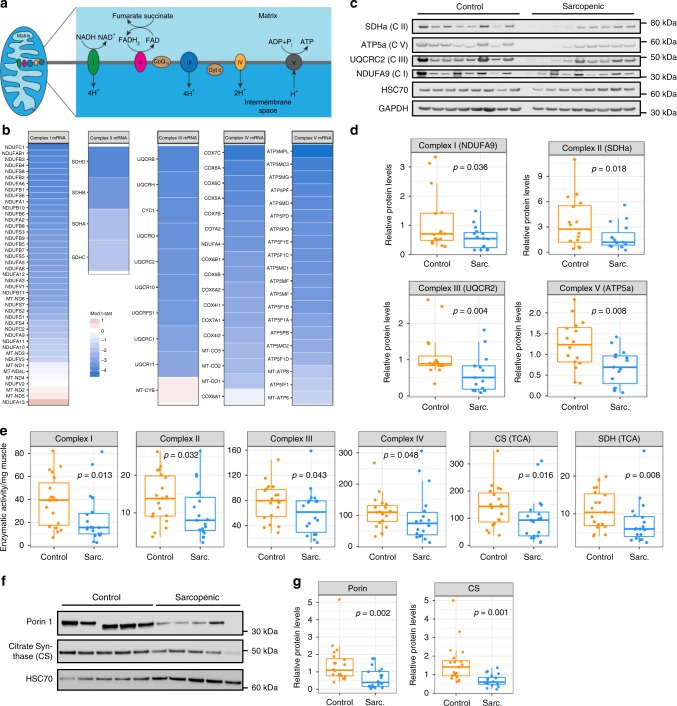
Mitochondrial bioenergetic activity is decreased in sarcopenic muscle. **a** Schematic representation of a mitochondrion and the five respiratory complexes of the electron transport chain. **b** Gene expression change in sarcopenic vs. control muscle of mRNAs encoding the subunits of the 5 mitochondrial respiratory chain complexes, color coded according to moderated t-statistics (SSS cohort; *n* = 19–20 per group). **c**, **d** Muscle protein expression of representative subunits of mitochondrial respiratory chain complexes and mitochondrial proteins measured by western blot. **c** Representative blots from one gel, with GAPDH and HSC70 included as house-keeping normalization controls. **d** Quantification of protein levels relative to GAPDH and HSC70 in all remaining samples analyzed (SSS cohort; *n* = 15–16 per group; *p* values calculated using Mann–Whitney tests). **e** Enzymatic activity per mg muscle tissue of mitochondrial complexes I–IV, citrate synthase (CS) and succinate dehydrogenase (SDH) measured on mitochondrial extracts from remaining muscle biopsies of control and sarcopenic participants (SSS cohort; *n* = 18 per group; *p* values calculated with Mann–Whitney tests). **f**, **g** Muscle protein expression of porin 1 and CS measured by western blot on remaining muscle samples of the SSS cohort (*n* = 20 per group; *p* values calculated with Mann–Whitney tests).

To further understand how the mitochondrial transcriptomic signature affects muscle bioenergetics during sarcopenia, the expression of all genes encoding the five mitochondrial respiratory complex subunits (Fig. 5a) was mapped in SSS sarcopenic vs. control muscle. Mitochondrial complex genes were downregulated in sarcopenic muscle across the five complexes (Fig. 5b), translating to a global reduction in the protein expression of active subunits of mitochondrial respiratory complexes (Fig. 5c). NDUFA9, SDHa, UQCR2, and ATP5a, representing complexes I, II, III, and V, respectively, were downregulated by 44–51% in sarcopenic muscle (Fig. 5d). Enzymatic assays on mitochondria isolated from muscle biopsies demonstrated functional deficits of mitochondrial activity in sarcopenia (Fig. 5e). In particular, the enzymatic activity of complexes I–IV was lower in sarcopenic muscle (Fig. 5e) and the activity of all complexes correlated positively with ALMi (Supplementary Fig. 3a). The activity of the two mitochondrial TCA cycle enzymes citrate synthase (CS) and succinate dehydrogenase (SDH) were strongly reduced in sarcopenic muscle (Fig. 5e), confirming that a global alteration of oxidative metabolism and energy production is perturbed in human sarcopenic muscle. Reduced complex activity was linked to lower amounts of mitochondria as it was not affected when normalized to CS activity (Supplementary Fig. 3b). The expression of other mitochondrial proteins such as CS and PORIN1 was also lower in sarcopenia (Fig. 5f, g), as expected from the downregulation of the ERRα/PGC1α/NRF1/TFAM network which controls both the amount and bioenergetic activity of mitochondria^31^. Thus, sarcopenia arises from a general bioenergetic deficit which differs mechanistically from primary mitochondrial myopathies caused by decreased activity of a specific complex. Nevertheless, the global bioenergetic deficit observed in sarcopenia can directly influence muscle contraction and performance which rely both on the amount and activity of mitochondria^2,3^.

### NAD^+^ levels are low in human sarcopenic muscle

**Fig. 6:**
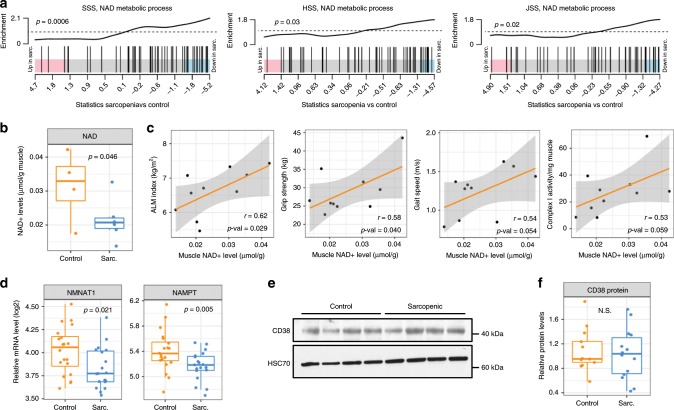
NAD^+^ levels are low in Sarcopenic muscle. **a** Gene-set enrichment plot of the “NAD metabolic process” (GO:0019674) on sarcopenic vs. control muscle mRNA in the discovery cohort (SSS, *n* = 19/20 per group) and 2 replication cohorts of different ethnicity (HSS, JSS; *n* = 4–28 per group as described in Supplementary Table 2). **b** NAD^+^ levels in remaining muscle biopsies of control and sarcopenic participants (SSS cohort, *n* = 4–6 per group; *p* values based on *t*-statistics). **c** Muscle NAD^+^ levels positively correlate to ALM-index, grip strength, gait speed, and mitochondrial complex I activity. Spearman rank correlation coefficient and its *p* value were calculated on *n* = 10 SSS muscle samples. **d** Muscle mRNA expression of NAD biosynthesis enzymes nicotinamide mononucleotide adenylyltransferase 1 (*NMNAT1*) and nicotinamide phosphoribosyltransferase (*NAMPT*) (SSS cohort; *n* = 19–20 per group; *p* values calculated with moderated *t*-statistics). **e**, **f** Muscle protein expression of CD38 by western blot on remaining muscle samples of SSS cohort (*n* = 13–14 per group; *p* values calculated with Mann–Whitney tests).

Intracellular levels of NAD^+^ have emerged as a major regulator of oxidative mitochondrial metabolism in health and disease^36^, leading to the possibility that they could contribute to the mitochondrial signature of sarcopenic muscle. In gene-set enrichment analysis, genes of the NAD metabolic process GO term were repressed in sarcopenia vs. control in SSS, HSS, and JSS (Fig. 6a). To confirm this signature, we measured NAD^+^ levels in remaining muscle biopsies of SSS as NAD^+^ levels can be detected on small amounts of muscle (Supplementary Fig. 4a) and are 99% correlated using biochemical and mass spectrometry assays (Supplementary Fig. 4a, b). Muscle NAD^+^ levels were decreased by 32% in sarcopenics vs. controls (Fig. 6b), and correlated positively with ALMi, grip strength, gait speed, and complex I activity (Fig. 6c). To understand the mechanisms underpinning reduced muscle NAD^+^ in sarcopenia, we analyzed the mRNA expression of the enzymes controlling NAD^+^ biosynthesis and salvage, and of NAD^+^ consuming enzymes (Supplementary Fig. 4c–f). Sarcopenic muscle had reduced expression of *NMNAT1* and *NAMPT*, two rate-limiting enzymes of NAD^+^ biosynthesis and salvage (Fig. 6d and Supplementary Fig. 4c). Similar trends were observed in the JSS cohort as well as in independent cohorts where sarcopenia was defined based on chronological age^37^ (Supplementary Fig. 5). In contrast, no increase in the expression of NAD^+^ consuming enzymes such as CD38 (Fig. 6e, f), PARPs (Supplementary Fig. 4e) or sirtuins (Supplementary Fig. 4f) was observed, suggesting that reduced NAD^+^ levels in human sarcopenia primarily result from inability to synthesize and recycle NAD^+^. Thus, human sarcopenia is driven by a major mitochondrial deficit detected both transcriptionally and functionally, for which reduced levels of NAD^+^ in muscle impair metabolic homeostasis and contribute to functional decline by altering muscle bioenergetics.

## Discussion

Aging is a multifactorial process with many overlapping physiological and molecular perturbations. Previous reports comparing skeletal muscle in elderly people and young adults have identified mechanisms that drive muscle aging without distinction for the mechanisms that specifically lead to pathological decline and physical disability^38–41^. With the recent recognition of sarcopenia as a specific pathological disorder under ICD-10 code M62.84 (ref. ^21^), a few candidate genes and pathways linked to pathological muscle aging have been deconvoluted through candidate-based approaches^42^. In the MEMOSA multicenter study, we report the first integrated molecular profile of human sarcopenia across ethnicities by comparing the muscle transcriptome in older people with physical disability to healthy controls of the same age group using genome-wide transcriptional profiling and functional validation. Our work establishes important advances in the mechanistic understanding of sarcopenia pathophysiology by characterizing the mechanisms leading to physical disability, and provides a unique resource for the identification of novel molecular targets and biomarkers to prevent sarcopenia and promote skeletal muscle health in older individuals.

The molecular signatures of sarcopenia vs. healthy older people revealed low mitochondrial bioenergetic capacity as the dominant signal in the transition from physiological to pathological muscle aging across ethnic groups. Oxidative phosphorylation and mitochondrial energy homeostasis were the most perturbed biological processes associated with sarcopenia in all ethnicities, with bioenergetic alterations spanning across all mitochondrial respiratory complexes both at the level of expression and activity. We also detected transcriptional signatures of altered mTOR signaling, translation, and protein synthesis in human sarcopenia, consistent with the role of anabolic resistance in the decline of muscle mass and strength during aging^33,34^. In contrast, neuromuscular dysfunction was not enriched in the transcriptional signature of sarcopenia while high inflammatory signaling was restricted to some but not all cohorts of human sarcopenia. These mechanisms described in preclinical muscle aging models^4,8,35^ may influence sarcopenia through nontranscriptional mechanisms or in patient subgroups, but our data point them away from primary hallmarks of human sarcopenia. The molecular footprints of sarcopenia in skeletal muscle were most strongly influenced by muscle mass and strength, with more modest contributions from gait speed, perhaps diluted by other mechanisms which influence this multifactorial measurement of mobility and quality of life. The ability to preserve high oxidative phosphorylation also positively associated with high muscle mass, grip strength, and gait speed as independent continuous analyses. Thus, our findings establish altered mitochondrial bioenergetic capacity through low oxidative phosphorylation as a strong and prominent determinant of the transition to disability in aged skeletal muscle.

At the molecular level, mitochondrial alterations in sarcopenia involve the perturbation of a transcriptional module with reduced expression or activity in sarcopenic muscle of the transcriptional regulators ERRα and NRF1 and their coactivator PGC-1α. These transcriptional regulators have been widely demonstrated to regulate mitochondrial gene expression in rodents and humans^30,31^. In particular, overexpression of PGC-1α or ERRα is sufficient to induce the expression of genes controlling mitochondrial function and to trigger functional benefits on oxidative phosphorylation and ATP generation. Thus, the downregulation of PGC-1α and ERRα target gene expression networks in sarcopenic muscle implies reduced transcriptional activity of ERRα and possibly of other PGC-1α-dependent transcription factors which may explain the global mitochondrial dysfunction observed in sarcopenia. Interestingly, perturbed NRF1 signaling was only detected through the transcriptional downregulation of its direct target genes, but its expression level was not affected by sarcopenia. In contrast, both the expression and transcriptional efficiency of PGC-1α and ERRα on their target promoters was reduced in sarcopenia. This observation is consistent with a priming function of low-PGC-1α/ERRα signaling in reducing oxidative phosphorylation and initiating pathological muscle decline as they act cooperatively through direct binding of PGC-1α to ERRα on target promoters and through a feed-forward loop where ERRα or PGC-1α activate the expression of their own promoters^31^. Translational mechanisms also overlapped with this transcriptional regulation as a large cluster of MRPs was downregulated in sarcopenia and likely contributed to the decreased protein expression of mitochondrial respiratory chain subunits. MRPs are also important to balance mitonuclear communication during aging and regulate a protective mitochondrial UPRmt important for the regulation of healthspan and longevity in preclinical models^32^. The expression of UPRmt genes (including mitochondrial heat-shock proteins, proteases and the downstream transcriptional response regulating mitohormesis from the nucleus) was low in sarcopenic muscle. Thus, inefficient UPRmt activation during sarcopenia fails to compensate the altered production of respiratory chain subunits and the damage to mitochondrial proteins induced by oxidative stress^10,17^. We also detected molecular signatures indicating altered mitochondrial dynamics through fusion and fission in sarcopenic muscle. The regulation of mitochondrial dynamics has been linked to the control of muscle metabolism and plasticity in preclinical models of muscle pathology and aging^11^. Interestingly, genetic loss of function of *Mfn2* and *Opa1* in mice is sufficient to cause sarcopenia^43–45^, suggesting that the down regulation we observed in sarcopenia could be causal in the loss of muscle mass and strength.

NAD^+^ decline during aging is well documented in preclinical models and has recently emerged in humans where skin and brain NAD^+^ decreases across the lifespan^46^. Although our NAD^+^ results require further validation as they were analyzed in a subset of individuals with sufficient amounts of remaining muscle biopsy from SSS, the reduction of skeletal muscle NAD^+^ levels in people with sarcopenia links for the first time NAD^+^ levels to an age-related pathology in humans and provides the first proof-of-principle of altered NAD^+^ biosynthesis in human skeletal muscle. Low amounts of mitochondria in human sarcopenia could potentially contribute to reduced NAD^+^ levels given the high mitochondrial NAD^+^ concentration, but mitochondria only represent 5–10% of myofiber volume^47^ and NAD^+^ is also abundant in larger cellular compartments^48^, which most likely also accounts for the sarcopenic NAD^+^ phenotype. Another possibility is that perturbed mitochondrial activity could induce the NAD^+^ depletion by altering metabolic fluxes. However, low NAD^+^ is a causal mechanism for mitochondrial perturbations in age-related pathologies in model organisms^49–51^. Reduced NAD^+^ levels in skeletal muscle of aged or Nampt deficient mice alter mitochondrial bioenergetics and impair muscle mass, strength and endurance^49,50,52,53^, suggesting that low NAD^+^ levels in sarcopenic people could directly contribute to impaired mitochondrial activity and sarcopenia progression in humans. Importantly, new therapeutic strategies have emerged to restore NAD^+^ levels in muscle by administration of dietary NAD^+^ precursors such as NR or NMN^46,53,54^, and the conversion of these precursors to NAD^+^ bypasses the rate limited enzyme NAMPT, which is downregulated in by sarcopenia. Combined with the demonstration that these interventions are safe and increase NAD^+^ levels in early human clinical testing^46,55,56^, our study creates the mechanistic basis to test the clinical efficacy of NAD^+^ precursors on muscle strength and physical function in older people with sarcopenia. More broadly, our work also highlights that nutritional and pharmacological mitochondrial therapeutics should be considered for the management of sarcopenia. This can be achieved by stabilizing the mitochondrial machinery by targeting cardiolipin^57^, promoting the elimination of damaged mitochondria by mitophagy with the natural molecule Urolithin A^18,58^, or targeting energy sensors like AMPK, PPARs, and sirtuins which converge on PGC-1α signaling^30^. The established benefits of physical activity on mitochondrial efficiency in aged skeletal muscle^2,47,59^ also suggest that exercise programs to improve sarcopenia should maximize mitochondrial adaptations that enhance bioenergetic coupling. Collectively, the MEMOSA study results provide a genome-wide molecular resource of mechanisms and biomarkers of pathological muscle aging across ethnicities. Our work establishes loss of mitochondrial oxidative capacity as a major mechanism of sarcopenia which can be assessed non-invasively using 31P imaging as a biomarker^60^, and provides a strong rationale for intervention trials targeting muscle mitochondrial bioenergetics to manage sarcopenia in older people.

## Methods

### Singapore sarcopenia study

Totally, 20 Chinese male participants aged 65–79 years and 20 control participants of the same age/ethnic group without a diagnosis of sarcopenia were recruited from two studies on healthy community-dwelling older men in Singapore (Singapore Sarcopenia Group and Aging in a Community Environment Study [ACES]). The National Healthcare Group Domain-Specific Research Board (NHG DSRB) approved the study, reference number 2014/01304, and each participant gave written informed consent. Self-reported ethnicity was collected during the inclusion visit and weight and height were measured to the nearest 0.1 kg and 1 cm, respectively. Total lean mass was measured through DXA scanning (APEX Software version 4.0.1, Discovery Wi DXA system). A standardized protocol was used to measure isometric hand grip with Jamar hand-held dynamometer and the mean of 3 three attempts from the dominant hand was used as the final measure. Gait speed was calculated from a timed 6-m walk. The diagnosis of sarcopenia was based on AWGSOP definition^61^ that was defined as the total appendicular lean mass normalized for height ≤ 7.00 kg m^−2^, evidence of either low physical performance based on gait speed ≤ 0.8 m s^−1^ OR low muscle strength based on hand grip < 26 kg. Semiopen muscle biopsies of the vastus lateralis muscle were collected using a BioPince^TM^ (Angiotech) 16 G full core biopsy needle with 3 adjustable stroke lengths (13 mm, 23 mm, and 33 mm) from the 20 male participants and 20 aged matched controls, snap frozen in liquid nitrogen and stored at −80 °C until further analysis.

### Hertfordshire sarcopenia study

Totally, 105 healthy community dwelling older men, 68–77 years old, who participated in the UK Hertfordshire Cohort Study were prospectively recruited the HSS as previously described^62^. Inclusion criteria for the HSS included the availability of birth records detailing birth weight and weight at 1 year. Men were excluded if they had a diagnosis of active ischemic heart disease, myopathy, or neuromuscular conditions affecting the legs or a history of diabetes. The Hertfordshire Research Ethics Committee approved the study under approval number 07/Q0204/68 and each participant gave written informed consent. Weight was measured once to the nearest 0.1 kg with floor scales (SECA, Hamburg, Germany). A total of 40 Caucasian participants from HSS were randomly selected for MEMOSA inclusion after stratification based on their sarcopenia phenotype using the first EWGSOP algorithm^22^. Height was measured to the nearest 0.1 cm. Total lean mass was calculated from body composition analysis by DXA (Hologic Discovery, software version 12.5). A standardized protocol was used to measure isometric grip strength with a Jamar dynamometer (Promedics, Blackburn, UK)^63^. Gait speed was calculated from a timed 3-m walk. The diagnosis of sarcopenia was based on the EWGSOP definition^22^ that was defined as the total appendicular lean mass normalized for height ≤ 7.23 kg m^−2^, evidence of either low physical performance based on gait speed ≤ 0.8 m s^−1^ or low muscle strength based on hand grip < 30 kg. Semiopen muscle biopsies with a Weil–Blakesley conchotome were obtained from participants after an overnight fast, as previously described^62^, snap frozen in liquid nitrogen, and stored at −80 °C until further analysis.

### Jamaica sarcopenia study

Totally, 40 male Afro-Caribbean participants aged 63–89 years were recruited through community-based (churches, community centers, senior citizen clubs) screening using the snowballing method for referrals. The University of West Indies Research Ethics Committee approved the study under approval number 180,10/11, and each participant gave written informed consent. All participants included in this study were from African origin based on self-report of at least three grandparents of African origin. Weight was measured once to the nearest 0.1 kg with floor scales (SECA, Hamburg, Germany). Height was measured to the nearest 0.1 cm with a stadiometer (SECA, Hamburg, Germany). Total lean mass was calculated from body composition analysis by DXA (GE Lunar Prodigy) Advance, Software: Encore 2011, Version 13.60.033). A standardized protocol was used to measure isometric grip strength with a Lafayette hand dynamometer (Lafayette Instrument Company, Lafayette, Indiana). Gait speed was calculated from a 6-min walk test. The diagnosis of sarcopenia was based on the EWGSOP definition^22^ as described above for HSS. Vastus lateralis muscle biopsies were obtained using a 5 mm Bergstrom needle (Stille-Werner, Ronkonkoma, NY) after participants had been characterized. Biopsies were obtained from the mid vastus lateralis muscle, about 12 cm above patella on the anterolateral thigh, following a 12 h overnight fast, and after 24–36 h of any structured exercise exposure. Muscle samples were separated, frozen in cooled isopentane, and stored at −80 °C until analysis.

### RNA extraction

Total RNA was extracted from muscle biopsies of HSS using the *mir*Vana miRNA Isolation Kit (Life Technologies) and from muscle biopsies of SSS and JSS using the QIAzol Lysis Reagent (Qiagen) followed by miRNAeasy purification Kit (Qiagen). Frozen muscle samples were placed into 600 µl Lysis/Binding buffer and homogenized using a Dispomix Homogenizer until all visible clumps were dispersed. The isolation procedure was then performed according to manufacturer’s instructions using the total RNA isolation protocol. RNA quantity was measured with Ribogreen (Life Technologies) and RNA quality was checked using the Standard Sensitivity RNA Analysis Kit on a Fragment Analyzer (Advanced Analytical Technologies). All RNA samples were homogeneous and passed quality control with 260/280 nm ratio > 1.8 and RIN scores > 7.

### RNA sequencing

For each sample, 250 ng of total RNA was employed as starting material for library preparation. Sequencing libraries were prepared using the TruSeq Stranded Total RNA HT kit with the Ribo-Zero Gold module (Illumina), followed by 13 cycles of PCR amplification with the KAPA HiFi HotStart ReadyMix (Kapa BioSystems). Libraries were quantified with Picogreen (Life Technologies) and size pattern was controlled with the DNA High Sensitivity Reagent kit on a LabChip GX (Perkin Elmer). Libraries were then pooled at an equimolar ratio and clustered at a concentration of 7 pM on paired-end sequencing flow cells (Illumina). Sequencing was performed for 2 × 101 cycles on a HiSeq 2500 (Illumina) with v3 chemistry. The generated data were demultiplexed using Casava. Reads were aligned to the human genome (hs_GRCh38.p2) using STAR^64^, and the number of reads mapped within genes was quantified by HTSeq^65^ (version HTSeq-0.6.1p1, mode union, strand reverse, quality alignment greater than 10). SSS samples had a sequencing depth of 75–104 million reads per sample, of which 34–77 million reads where uniquely mapped. HSS and JSS had a sequencing depth of 51–110 and 55–88 million reads per sample, respectively, out of which 38–84 million and 39–69 million were uniquely mapped.

### mRNA differential expression & pathway enrichment analyses

All statistical analyses data were performed using R version 3.3.3 and relevant Bioconductor packages (e.g., limma 3.30.13, edgeR 3.16.5). Unless otherwise stated, 40 samples from each cohort were analyzed. For differential expression analysis, all samples with more than 35 million uniquely mapped reads were included. One sample (from the SSS cohort) which did not reach this threshold because of an abnormally low percentage of uniquely mapped reads was excluded from the analysis. Differentially expressed genes between control and sarcopenic samples were defined using the limma package^66^. Briefly, after removing genes with a mean expression lower than 20 reads, data were normalized by the trimmed mean of M-values method as implemented in the edgeR function calcNormFactors^67^, and the voomWithQualityWeights function was applied to model the mean-variance relationship and estimate the sample-specific quality weights^68^. *p* Values were corrected for multiple testing using the Benjamini–Hochberg method. The same procedure was applied when characterizing the associations between gene expression and the continuous or categorical parameters (ALMi, grip strength and walking speed) used to define sarcopenia. Pathway enrichment analysis was performed using CAMERA^69^, a competitive gene set test querying whether a set of genes annotated in the Molecular Signatures Database (MSigDB)^70^ is enriched in differentially expressed or continuously associated genes. MSigDB (http://software.broadinstitute.org/gsea/msigdb/index.jsp) v5.2 collections H (hallmark gene sets), C2 (curated gene sets), and C5 (GO gene sets) were used to perform pathway analyses. To circumvent the absence of a mammalian UPRmt GO category, we have created a custom mammalian UPRmt gene set using the lower organism UPRmt GO:0034514 category and manual curation of the mammalian UPRmt homologs based on published reviews^17,71^. To circumvent the redundancy of several gene sets in public databases, we removed gene sets with a gene overlap >75% from figure visualization (although these gene sets were still considered for computation of FDRs). The overlap between 2 gene sets of different sizes was defined as OL = 2 × *c*/(*n* + *m*) × 100, where *n* and *m* are the size of the 2 gene sets and *c* is the genes in common, and the threshold of 75% was selected from a range from 50 to 90% to maximize biological diversity while minimizing overlaps).

### Network and gene ontology analyses

Protein interaction networks were generated with the 149 protein coding genes differentially regulated in sarcopenia using STRING version 10 (http://string-db.org/), using all data sources, a confidence score of 0.9, and the maximum number of interactors shown in the first shell set to 5. The interaction network was colored manually based on biological function of the proteins and the network connectivity was enriched at a *p* value < 1.0e−16 when compared to a random sampling of 149 proteins. In addition, the unique identifiers of differentially expressed genes were used as an input for functional analyses using Cytoscape (version 3.5.1). Genes differentially expressed were used for functional enrichment analysis to decipher functionally grouped gene ontology and biological process using ClueGO. pV correction was estimated using a Bonferroni stepdown method. Results are presented as pie charts in Fig. 1e.

### Transcription factor binding enrichment analysis

Molecular Signature Database (MsigDB, http://software.broadinstitute.org/gsea/msigdb/), was used to identify the transcription factor target gene sets significantly associated with the 179 differentially expressed genes identified in Fig. 1a at an FDR < 0.05. Under MsigDB, the C3 subcollection transcription factor targets containing 674 motif gene sets was used for this analysis. To investigate the enrichment of ERRA and NRF1 binding sites in distal and proximal regulatory regions of the 178 genes (excluding chr M) associated with sarcopenia, we extracted their DNA sequence via UCSC table browser using the hg38 human genome assembly. Sequence lengths interrogated included 5 kb and 5–20 kb upstream/downstream of the transcriptional starting site. Transcription factor binding site/motif enrichment analysis was performed as previously described^72^ using the findMotifs.pl tool embedded within Homer, a tool used for motif discovery and next generation sequencing analysis. Input sequences were randomly scrambled and used as background sequences for enrichment analysis. To ensure robustness of results, hypergeometric test was repeated 1000 times to compute Benjamini–Hochberg corrected median adjusted *p* value/*q*-values.

### Gene expression validation by nanoString nCounter

mRNA expression of 70 genes of interest selected based on upregulation or downregulation in sarcopenic muscle or on biological relevance was validated using a customized nanoString nCounter panel (Supplementary Data 5), a method orthogonal to sequencing, based on the binding of probes directly to the mRNA. Each target gene was detected using a pair of reporter and capture probes. Reporter probes carry a unique color code that enables the molecular barcoding of the genes of interest. The expression level of a gene is measured by counting the number of times the color-coded barcode for that gene is detected. The experiment was performed from 100 ng total RNA, strictly following the manufacturer’s recommendations. Ten genes, stably expressed in skeletal muscle, were selected based on their low coefficient of variation in the RNAseq profiles of this study, and used as housekeeping genes for normalization. Primary analysis was performed with the dedicated nSolver software (nanoString).

### Mitochondrial enzymatic activity

Mitochondrial enzyme and respiratory chain complex activities were measured on mitochondrial fractions isolated from 15 to 30 mg of frozen muscle biopsies as previously described^73,74^, for all biopsies where sufficient material remained after transcriptomic experiments. Briefly, tissues were homogenized in 10 mM potassium phosphate buffer (pH 7.4) and mitochondrial-enriched fractions collected after a 800*g* centrifugation were used for enzymatic assays. Complex I activity was assessed by measuring rotenone-sensitive coenzyme Q1-dependent NADH reduction. SDH and complex II activities were assessed by measuring 2,6-dichlorophenolindophenol (DCPIP) reduction either in the absence or in presence of coenzyme Q, respectively. Complex III activity was measured by cytochrome C reduction. Complex IV activity was measured by assessing cytochrome C oxidation. Citrate synthase activity was assessed by measuring DTNB reduction at 412 nm in the presence of Acetyl-CoA and oxaloacetate. Enzymatic activities were normalized to the amount of muscle analyzed for all samples with remaining biopsy material (*n* = 38).

### Western blot

Western blots were performed using protein extracts remaining from the enzymatic assay preparations. Protein concentration was determined by a bicinchoninic acid (BCA) assay (Pierce #23227) and samples were prepared with 4x LDS sample buffer (Novex #NP007). 30 μg protein per sample were resolved by standard western blot procedure on 4–12% bis–tris protein gels (Novex #WG1403BX10), and then transferred to PVDF membranes using the semi-dry system from Life Technologies/Invitrogen. Membranes were cut based on molecular ladder size to detect proteins of different size from the same membrane with different antibodies. Detection was achieved using an OXPHOS antibody cocktail (Abcam #ab110412, 1:1000), or antibodies against Porin1 (Abcam #ab15895, 1:1000), citrate synthase (Abcam #ab96600, 1:1000), CD38 (R&D System #MAB24041, 2 μg ml^−1^), GAPDH (Abcam #ab37168, 1:5000) and HSC70 (Santa Cruz, sc-7298; 1:50000), with the relevant secondary antibodies and using enhanced chemiluminescence (ECL; Pierce #321016) detected by standard autoradiography with exposure time adjusted to the expression of each protein. Complexes I–III and V were correctly detected in all samples but complex IV was only detected in less than 20% of samples and was excluded from the analysis. Films were scanned and quantified using Image J.

### NAD^+^ quantification

NAD^+^ levels were measured in human muscle biopsies of the MEMOSA study as previously described^75^, for all biopsies where sufficient material remained after transcriptomic experiments. Briefly, 5 mg muscle tissue from remaining biopsies was lysed in 200 μL 0.6 M perchloric acid and the supernatant was diluted 250-fold in 100 mM Na_2_HPO_4_ pH 8.0. 100 μL of diluted sample was combined with 100 μL reaction mix (100 mM Na_2_HPO_4_ pH 8, 2% ethanol, 90 U mL^−1^ alcohol dehydrogenase, 130 mU mL^−1^ diaphorase, 10 μM resazurin, 10 μM flavin mononucleotide, 10 mM nicotinamide), and the fluorescence increase (Ex 540 nm/Em 580) was measured over 10 min. NAD^+^ content was calculated from a standard curve and normalized to tissue weight.

For optimization of NAD^+^ measurements and benchmarking using mass spectrometry, frozen muscle biopsies (~2–50 mg) independent of the study were extracted in 1300 µL of a cold mixture of methanol:water:chloroform in 5:3:5 (v/v). Extracts were spiked with 60 µL of [U]−13C-NAD^+^ labeled biomass from home-made yeast as internal standard, and kept cold throughout the procedure. Muscle extracts were homogenized with 3 mm tungsten carbide beads using a tissue mixer (Qiagen TissueLyser II) for 3 min at 20 Hz, followed by 20 min 1500 rpm shaking at 4 °C in a thermo-shaker (Thermomixer C, Eppendorf). Samples were then centrifuged 10 min 21,000 × *g* at 4 °C, and the upper polar phase was dried overnight in a vacuum centrifuge at 4 °C and 5 mbar, and then stored at −80 °C, before analysis. Dry samples were reconstituted in 50 µL 60% (v/v) acetonitrile:water, centrifuged for 2 min at 21,000 × *g*, and the supernatant was transferred into a glass vial for hydrophilic interaction ultra high performance liquid chromatography mass spectrometry (UHPLC-MS) analysis, in a randomized order. The UHPLC consisted of a binary pump, a cooled autosampler, and a column oven (DIONEX Ultimate 3000 UHPLC + Focused, Thermo Scientific), connected to a triple quadrupole spectrometer (TSQ Vantage, Thermo Scientific) equipped with a heated electrospray ionization (H-ESI) source. Two microlitre of each sample were injected into the analytical column (2.1 mm × 150 mm, 5 µm pore size, 200 Å HILICON iHILIC^®^-Fusion(P)), guarded by a precolumn (2.1 mm × 20 mm, 200 Å HILICON iHILIC^®^-Fusion(P) Guard Kit) operating at 35 °C. The mobile phase (10 mM ammonium acetate at pH 9, A, and acetonitrile, B) was pumped at 0.25 mL min^−1^ flow rate over a linear gradient of decreasing organic solvent (0.5–16 min, 90–25% B), followed by re-equilibration for a total run time of 30 min. The MS operated in positive mode at 3500 V with multiple reaction monitoring (MRM) and in each scan event: scan width was 1 m/z and scan time was 0.05 s, peak with for Q1 was 0.25 FWHM and for Q3 0.70 FWHM. The sheath gas was 20 arbitrary units, and the auxiliary gas was kept 15 arbitrary units. The temperature of vaporizer was 280 °C and the temperature of the ion transfer tube was 310 °C. The tube lens voltage and collision energy were individually optimized for each fragment ion of NAD^+^ (664 > 428, 524). The software Xcalibur v4.1.31.9 (Thermo Scientific) was used for instrument control, data acquisition and processing. Positive ion mode extracted chromatograms using the MRM trace of NAD^+^ were integrated. A calibration curve of NAD^+^ (Sigma) with 10 data points between 0.62 and 40 µM was used for quantification of NAD^+^ in biological samples, normalized to internal standard. Dehydrated polar extracts were independently reextracted for enzymatic NAD^+^ quantification using perchloric acid extraction as described above.

### Data representation and statistics

Statistical methods for transcriptomic experiments using two-tailed statistics and correction for multiple testing are reported above. Data distributions were plotted as box-plots representing the 25th percentile (1Q), the median, and 75th percentile (3Q), with whiskers extending from the 1Q to the smallest value within 1.5*interquartile range (IQ = 3Q−1Q) and from the 3Q to larger value within 1.5*IQ. Associations between two continuous variables were determined using Spearman rank correlations. Functional validation of hypotheses on mitochondrial function generated from the transcriptomic results were analyzed using one-tailed nonparametric Wilcoxon/Mann–Whitney tests for mitochondrial complex expression and activity, or parametric *t* statistics for NAD^+^ results, after assessing the distribution of the variables with a Shapiro–Wilk normality test.

### Reporting summary

Further information on research design is available in the Nature Research Reporting Summary linked to this article.

## Supplementary information

Supplementary Information New

Description of Additional Supplementary Files

Supplementary Dataset 1

Supplementary Dataset 2

Supplementary Dataset 3

Supplementary Dataset 4

Supplementary Dataset 5

Reporting Summary

## Data Availability

The unprocessed transcriptomic data of this study have been deposited in the Gene Expression Omnibus (https://www.ncbi.nlm.nih.gov/geo/) under accession numbers GSE111006, GSE111010, GSE111016, and integrated in the series GSE111017. The individual data points of all box plot figures are provided as Source Data. Other datasets analyzed during the current study are available from the corresponding authors on reasonable request. Due to ethical concerns, supporting clinical data cannot be made openly available. The MEMOSA team can provide the data on request subject to appropriate approvals, after a formal application to the Oversight Group of the different cohorts through their respective corresponding author.

## Source data

Source Data New
