# Supplementary material for: Mitochondrial oxidative capacity and NAD+ biosynthesis are reduced in human sarcopenia across ethnicities
Source: Nat Commun. 2019 Dec 20;10:5808. doi: 10.1038/s41467-019-13694-1 (PMC6925228; doi:10.1038/s41467-019-13694-1)
Supplement: Supplementary file 1 — Supplementary Information New [file 41467_2019_13694_MOESM1_ESM.pdf]

SUPPLEMENTARY TABLE 1. Participant Characteristics.

	SSS (n=40*) Mean +/- SD	HSS (n=40) Mean +/- SD	JSS (n=40**) Mean +/- SD
Ethnicity	Chinese	Caucasian	Afro-Caribbean
Gender	Male	Male	Male
Age (years)	71.48 +/- 4.28	72.93 +/- 2.44	75.28 +/- 7.70
Height (m)	1.65 +/- 0.06	1.72 +/- 0.06	1.70 +/- 0.08
Weight (kg)	60.34 +/- 6.33	80.63 +/- 13.69	61.94 +/- 13.91
BMI (kg/m^2)	22.08 +/- 1.43	27.29 +/- 3.62	21.47 +/- 4.35
Total Lean body mass (DXA) (kg)	41.44 +/- 4.63	54.61 +/- 7.22	47.05 +/- 6.29
Fat Mass (DXA) (kg)	15.56 +/- 3.80	22.10 +/- 7.25	11.45 +/- 9.32
ALMi (kg/m^2)	6.81 +/- 0.75	7.93 +/- 0.90	7.48 +/- 0.81
Gait speed (m/s)	1.24 +/- 0.30	1.10 +/- 0.19	1.05 +/- 0.26
Grip strength (kg)	30.01 +/- 6.86	37.08 +/- 7.17	30 +/- 8.64

SSS: Singapore Sarcopenia Study, HSS: Hertfordshire Sarcopenia Study, JSS: Jamaica Sarcopenia Study.
BMI: Body mass Index, m/s: metres per second, ALMi: appendicular lean mass index.
*: one sample was discarded from RNAseq data analyses after quality control (fraction of uniquely mapped reads < 0.5)
**: one sample was excluded because of inadequate amount of RNA and RNAseq was not performed

SUPPLEMENTARY TABLE 2. Case/control stratification of clinical variables in the 3 cohorts.

SSS	Control[1] (n=20) Mean	+/-	SD	Sarcopenic[2] (n=20) Mean	+/-	SD	p-value
Age (years)	70.20	+/-	4.10	72.75	+/-	4.18	N.S.
BMI (kg/m^2)	22.70	+/-	1.01	21.47	+/-	1.54	5.3E-03
Lean body mass (kg)	44.53	+/-	3.86	38.36	+/-	3.01	2.2E-06
Fat Mass (kg)	15.39	+/-	3.67	15.73	+/-	4.02	N.S.
ALMi (kg/m^2)	7.42	+/-	0.39	6.20	+/-	0.48	1.4E-08
Grip strength (kg)	35.11	+/-	5.93	24.91	+/-	2.56	2.0E-08
Gait speed (m/s)	1.40	+/-	0.23	1.09	+/-	0.29	6.3E-04

HSS	Control[3] (n=28)			Low Muscle Mass[4] (n=9)				Low Muscle Function[5] (n=7)				Sarcopenic[6] (n=4)			
	Mean	+/-	SD	Mean	+/-	SD	p-value	Mean	+/-	SD	p-value	Mean	+/-	SD	p-value
Age (years)	72.60	+/-	2.34	73.03	+/-	2.67	NS	74.91	+/-	2.69	NS	74.28	+/-	3.59	NS
BMI (kg/m^2)	27.93	+/-	3.84	24.89	+/-	1.71	2.5E-03	27.31	+/-	2.44	NS	26.43	+/-	1.37	NS
Lean mass (kg)	56.68	+/-	6.55	46.77	+/-	3.47	3.4E-06	51.52	+/-	7.96	NS	46.13	+/-	4.27	6.9E-03
Fat Mass (kg)	22.83	+/-	7.80	19.77	+/-	4.74	NS	22.94	+/-	6.05	NS	23.41	+/-	4.37	NS
ALMi (kg/m^2)	8.24	+/-	0.78	6.82	+/-	0.26	8.2E-10	7.44	+/-	0.95	NS	6.74	+/-	0.25	1.9E-06
Grip strength (kg)	39.93	+/-	5.12	30.78	+/-	6.74	3.2E-03	27.14	+/-	7.38	2.9E-03	25.50	+/-	6.61	0.019
Gait speed (m/s)	1.15	+/-	0.14	1.00	+/-	0.25	NS	0.92	+/-	0.28	NS	0.91	+/-	0.36	NS

JSS	Control[3] (n=15)			Low Muscle Mass[4] (n=14)				Low Muscle Function[5] (n=20)				Sarcopenic[6] (n=9)			
	Mean	+/-	SD	Mean	+/-	SD	p-value	Mean	+/-	SD	p-value	Mean	+/-	SD	p-value
Age (years)	75.60	+/-	7.81	76.64	+/-	7.10	NS	74.75	+/-	8.23	NS	76.78	+/-	7.85	NS
BMI (kg/m^2)	22.72	+/-	3.60	17.94	+/-	1.96	1.9E-04	21.57	+/-	4.82	NS	18.29	+/-	2.23	1.2E-03
Lean mass (kg)	49.54	+/-	7.42	42.62	+/-	3.82	4.4E-03	46.03	+/-	5.51	NS	42.04	+/-	4.44	5.2E-03
Fat Mass (kg)	12.4	+/-	10.4	6.30	+/-	5.18	NS	12.46	+/-	9.10	NS	7.28	+/-	6.09	NS
ALMi (kg/m^2)	8.07	+/-	0.62	6.66	+/-	0.37	1.1E-07	7.29	+/-	0.85	3.5E-03	6.58	+/-	0.42	5.6E-07
Grip strength (kg)	36.47	+/-	8.44	26.14	+/-	6.84	1.2E-03	24.60	+/-	5.92	1.0E-04	22.78	+/-	6.24	1.80E-04
Gait speed (m/s)	1.15	+/-	0.21	1.03	+/-	0.28	NS	0.95	+/-	0.28	0.021	0.98	+/-	0.30	NS

1: ALMi>7kg/m^2 and GripStrength≥26kg and GaitSpeed>0.8m/s

2: ALMi≤7kg/m^2 and (GripStrength>26kg or GaitSpeed≤0.8m/s) - AWGSOP consensus definition[1]

3: ALMi>7.23kg/m^2 and GripStrength>30kg and GaitSpeed>0.8m/s

4: ALMi≤7.23kg/m^2

5: GripStrength≤30kg or GaitSpeed≤0.8m/s

6: ALMi≤7.23kg/m^2 and (GripStrength≤30kg or GaitSpeed≤0.8m/s) – EWGSOP consensus definition[2]

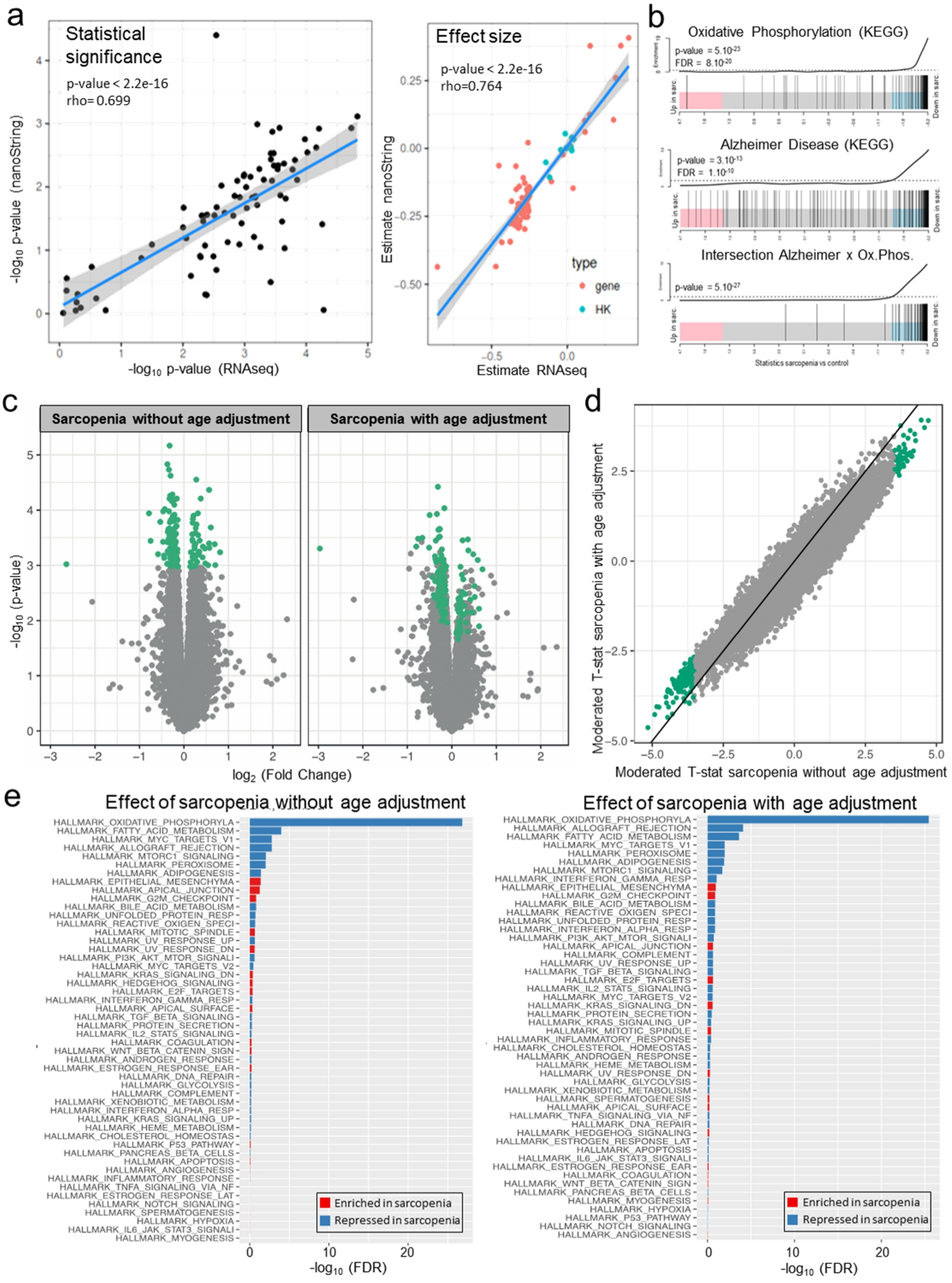

Supplementary Figure 1. Mitochondrial dysfunction is the major transcriptional signature of human sarcopenic muscle after age adjustment and with independent technical replication. (a) Correlation of RNA sequencing results with independent nanoString nCounter validation for the 80 genes measured with both technologies in human skeletal muscle (n=39 muscle samples from SSS participants). Spearman rank correlation coefficient and p-value are reported**. (b)** CAMERA gene set enrichment plot of sarcopenic vs control muscle in SSS for KEGG oxidative phosphorylation (Kegg hsa00190; MSigDB M19540), Alzheimer's disease (Kegg hsa05010; MSigDB M16024), and their intersection. The intersection of the 2 gene sets was extracted to demonstrate that the overlap between the transcriptional signatures of neurodegeneration and sarcopenia is driven by oxidative phosphorylation genes. **(c)** Volcano plot of differentially expressed genes in sarcopenic vs age-matched muscle for models without and with age adjustment as a covariate. p-values were calculated using moderated t-statistic. Genes with a FDR lower than 10% in the model without age adjustment are represented in green in both plots. **(d)** Scatter plot of the moderated t-values for each gene in the models without and with age adjustment. Genes with a FDR lower than 10% in the model without age adjustment are represented in green and the identity line is represented in black. **(e)** Gene set enrichment analysis of sarcopenic vs control muscle using models without and with age adjustment as a covariate, performed using CAMERA and the Hallmark gene set collection from MSigDB. Gene sets are ordered according to the significance of their enrichment. For all panels, n=19/20 muscle samples per group from SSS participants.

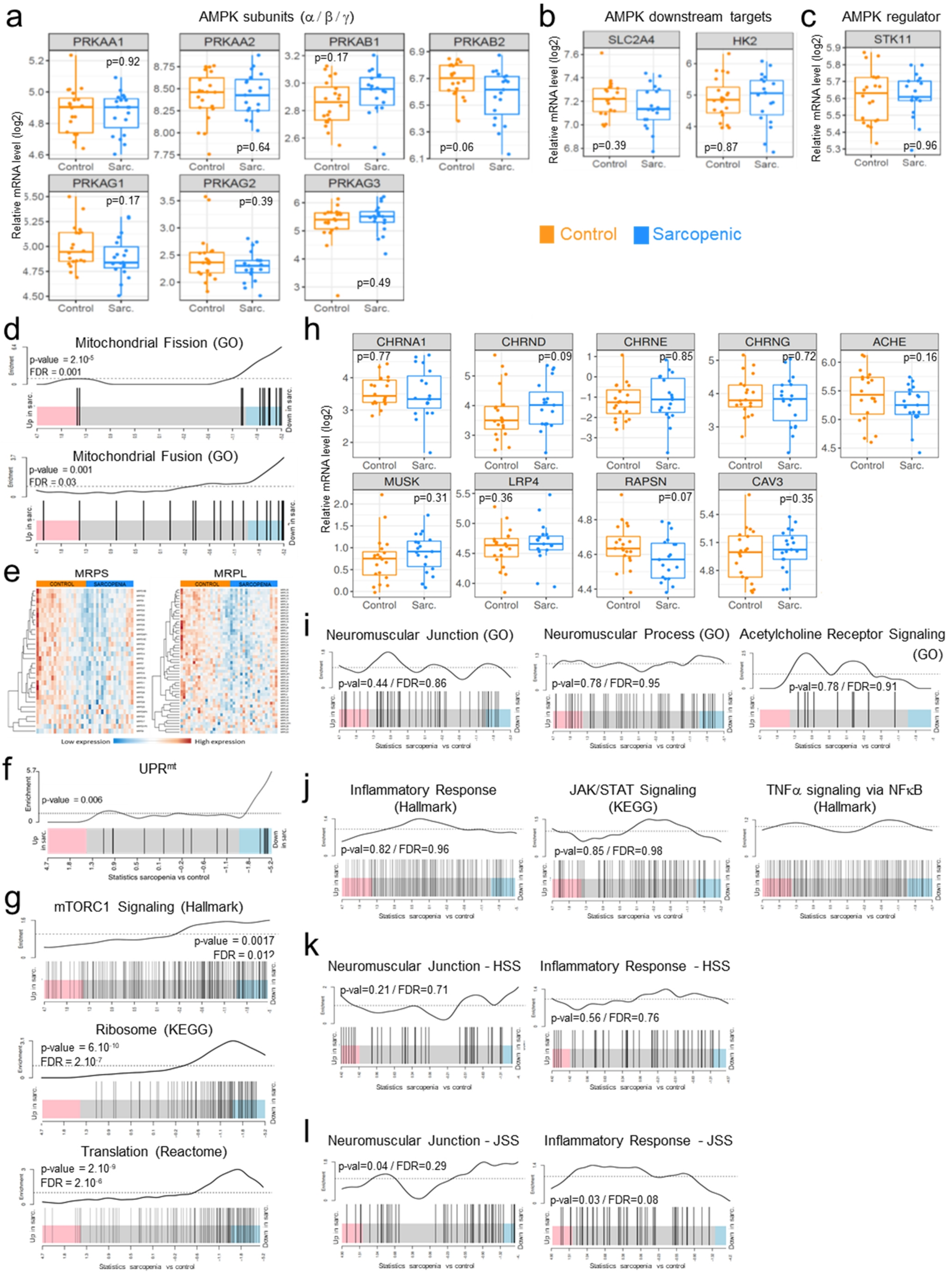

Supplementary Figure 2. Muscle RNAseq reveals transcriptional changes in sarcopenia linked to mitochondrial function and protein synthesis, but does not detect transcriptional changes linked to neuromuscular dysfunction, denervation and inflammation. (a-c) mRNA expression of AMP-activated Protein Kinase (AMPK) subunits (a), of its downstream targets Glut4/SLC2A4 and Hexokinase 2 (HK2) (b), and of its upstream regulator LKB1/STK11 (c), in SSS sarcopenic vs control muscle. **(d;f-g;i-j)** Gene set enrichment plots of sarcopenic vs control muscle in SSS for mitochondrial dynamics (d; GO:0000266 & GO:0008053), UPRmt (f), muscle protein synthesis (g; MSigDB M5924, M189 & M8229), neuromuscular function (I; GO:0007528, GO:0050905, & GO:0095500) and inflammation (j; MSigDB M5932, M17411& M5890). For panel f, a custom mammalian UPRmt gene set was used (see methods). **(e)** Heat maps of all genes coding small (MRPS) and large (MRPL) subunits of the mitochondrial ribosome in sarcopenic vs control muscle. **(h)** mRNA expression of genes controlling neuromuscular junctions in SSS sarcopenic vs control muscle. (**k-l)** Neuromuscular junction and inflammatory response gene set enrichment plots of sarcopenic vs control muscle in HSS (k; n=4/28 HSS muscle samples per group) and in JSS (l; n=9/15 HSS muscle samples per group). In a-j, n=19/20 SSS muscle samples per group. (a-c;h) Nominal moderated t-test p-values (d;f-g;i-l) CAMERA p-values and adjusted p-values (FDR).

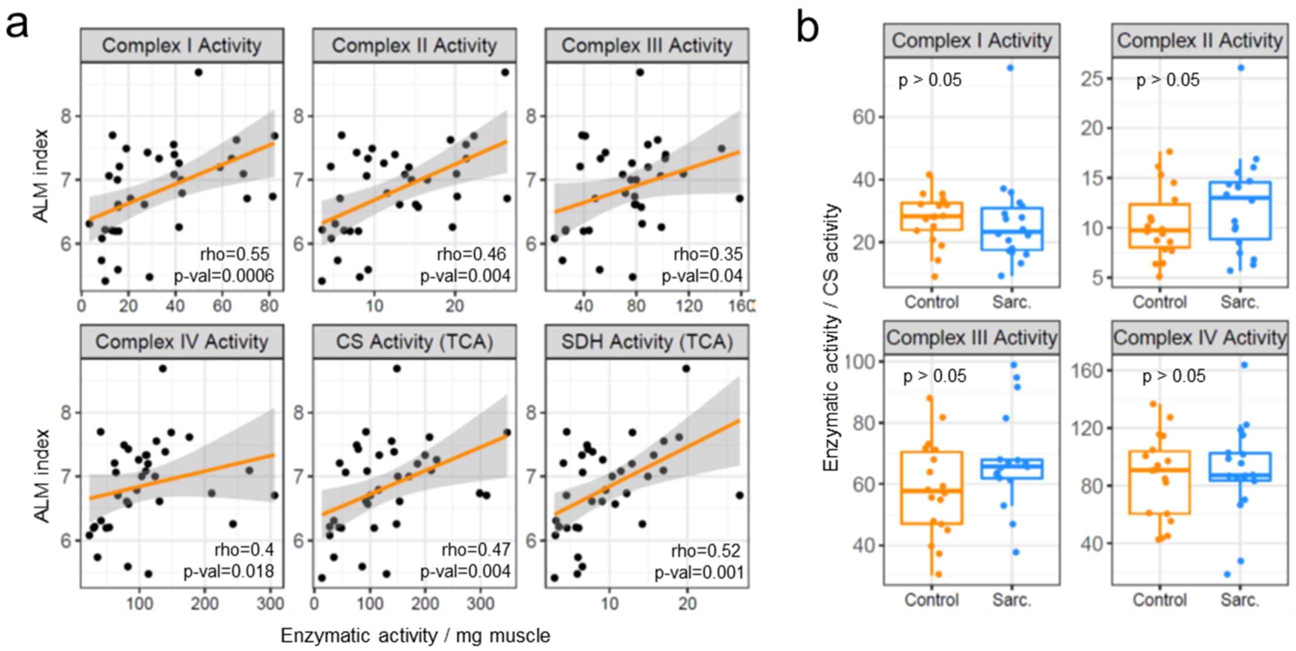

Supplementary Figure 3. Mitochondrial activity in case/control and continuous measures of sarcopenia. (a) Mitochondrial enzymatic activities per mg of muscle are correlated to ALM index. Regression lines are represented in orange and their 95% confidence intervals in gray. n=36 SSS muscle samples. **(b)** Enzymatic activity of mitochondrial complexes I, II, III and IV normalized to Citrate Synthase (CS) on mitochondrial extracts from muscle biopsies of control and sarcopenic participants. n=18 SSS muscle samples per group.

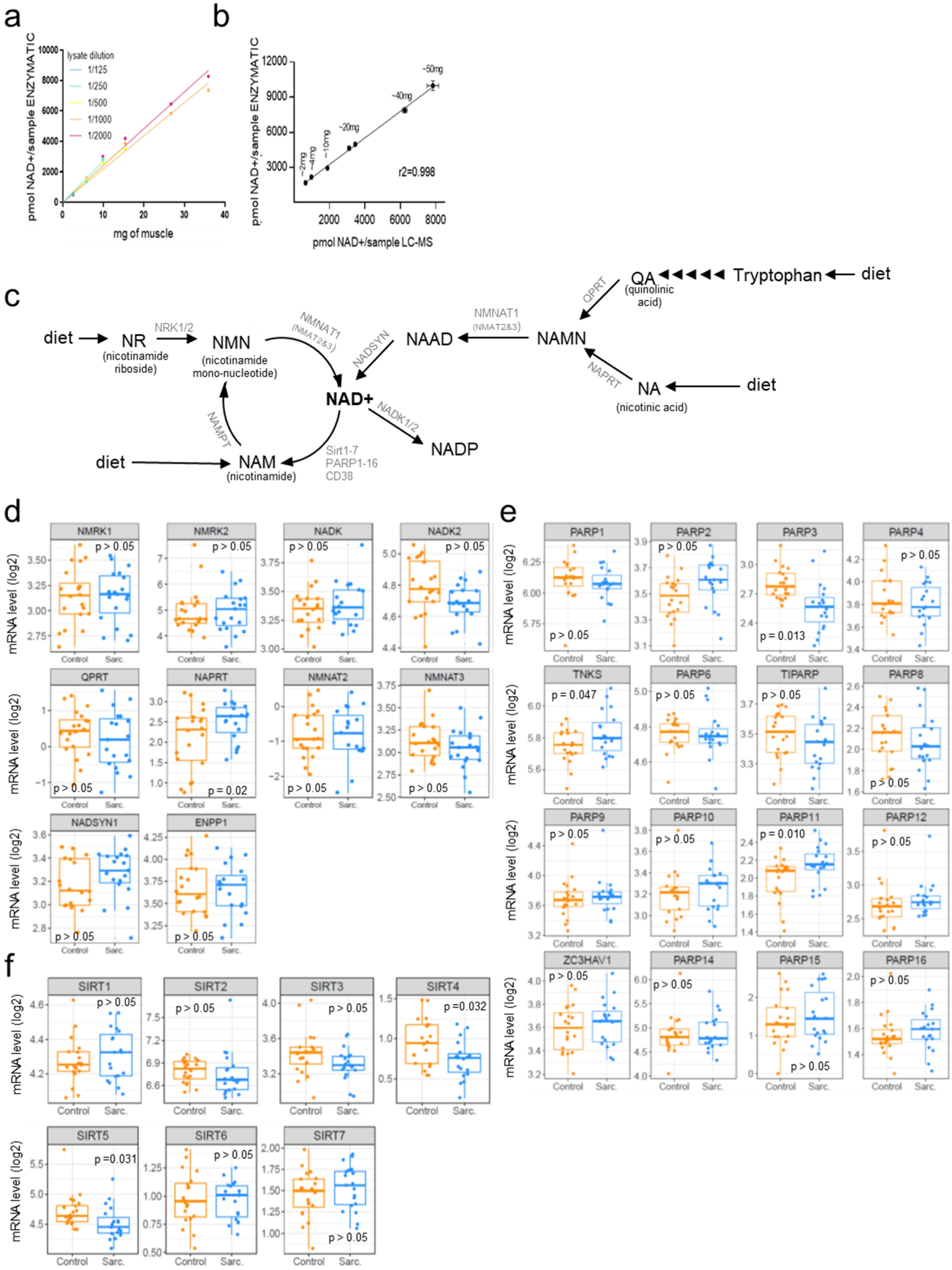

Supplementary Figure 4. NAD pathway in case/control and continuous measures of sarcopenia. (a) The enzymatic quantification of NAD+ is linear with different amounts muscle biopsy and different dilutions of muscle extracts. n=3 independent biopsies. **(b)** Enzymatic quantification of NAD+ tightly correlates to quantification of NAD+ by liquid chromatography – mass spectrometry (LC-MS) on the same muscle extracts. n=3 independent biopsies. **(c)** NAD+ biosynthesis pathway. **(d-f)** mRNA expression of NAD biosynthesis genes (d), PARPs (e) and sirtuin (SIRT, f) genes in SSS sarcopenic vs control muscle. Nominal moderated t-test p-values are reported using n=19/20 SSS muscle samples per group.

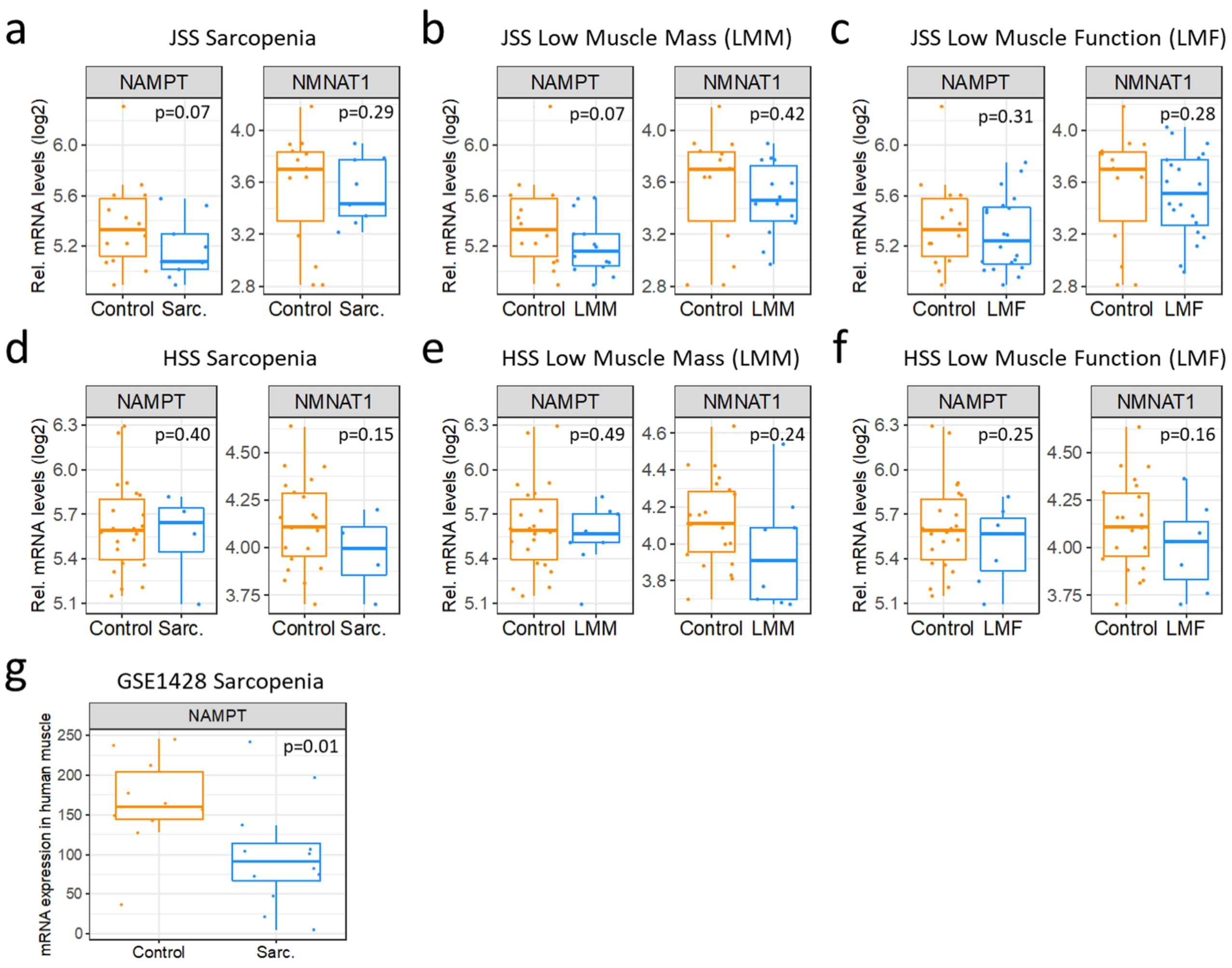

Supplementary Figure 5. The transcriptional downregulation of *Nampt* is observed in other human cohorts of sarcopenia. Muscle *Nampt* mRNA expression was analysed in Sarcopenia vs control in JSS (a) and HSS cohorts (d); low appendicular lean mass index vs control in JSS (b) and HSS (e) cohorts; and low muscle function (grip strength or gait speed) vs control in JSS (c) and HSS (d) cohorts. (g) Muscle *Nampt* mRNA expression was analysed from the public sarcopenia dataset (GEO entry GSE1428) where sarcopenia was described according to chronological age. P-values are based on a one sided Mann-Whitney test with n=24-35 (JSS; a-c) and n=32-35 (HSS; d-f) muscle samples per cohort stratified in the different phenotypes as described in Supplementary Table 2, and n=10-12 muscle samples per group (g).

SUPPLEMENTARY REFERENCES

1. Chen, L.K., *et al.* Sarcopenia in Asia: consensus report of the Asian Working Group for Sarcopenia. *J Am Med Dir Assoc* **15**, 95-101 (2014).

2. Cruz-Jentoft, A.J., *et al.* Sarcopenia: European consensus on definition and diagnosis: Report of the European Working Group on Sarcopenia in Older People. *Age Ageing* **39**, 412-423 (2010).
