# Supplementary material for: Mitochondrial oxidative capacity and NAD+ biosynthesis are reduced in human sarcopenia across ethnicities
Source: Nat Commun. 2019 Dec 20;10:5808. doi: 10.1038/s41467-019-13694-1 (PMC6925228; doi:10.1038/s41467-019-13694-1)
Supplement: Supplementary file 2 — Description of Additional Supplementary Files [file 41467_2019_13694_MOESM2_ESM.pdf]

**Title:** Supplementary Data 1.
**Description:** Table of RNAseq quality control in the SSS, HSS and JSS cohorts.

**Title:** Supplementary Data 2.
**Description:** List of individual gene expression by RNAseq in SSS. Different tabs represent the sarcopenic vs control differential expression, and the continuous association of gene expression with ALMi, grip strength and walking speed.

**Title:** Supplementary Data 3.
**Description:** List of nanoString vs RNAseq gene expression in SSS for genes selected for validation.

**Title:** Supplementary Data 4.
**Description:** List of individual gene set enrichment in the SSS pathway enrichment analysis.Different tabs represent the sarcopenic vs control enrichment using CAMERA, and the enrichment of the genes correlated with ALMi, grip strength and walking speed.

**Title:** Supplementary Data 5.
**Description:** NanoStringTargetSequences
