# Supplementary material for: Mitochondrial oxidative capacity and NAD+ biosynthesis are reduced in human sarcopenia across ethnicities
Source: Nat Commun. 2019 Dec 20;10:5808. doi: 10.1038/s41467-019-13694-1 (PMC6925228; doi:10.1038/s41467-019-13694-1)
Supplement: Supplementary file 8 — Reporting Summary [file 41467_2019_13694_MOESM8_ESM.pdf]

nature research

Corresponding author(s): FEIGE

Reporting Summary

Nature Research wishes to improve the reproducibility of the work that we publish. This form provides structure for consistency and transparency in reporting. For further information on Nature Research policies, see Authors & Referees and the Editorial Policy Checklist.

Statistical parameters

When statistical analyses are reported, confirm that the following items are present in the relevant location (e.g. figure legend, table legend, main text, or Methods section).

n/a	Confirmed	
☐	☒	The exact sample size (n) for each experimental group/condition, given as a discrete number and unit of measurement
☐	☒	An indication of whether measurements were taken from distinct samples or whether the same sample was measured repeatedly
☐	☒	The statistical test(s) used AND whether they are one- or two-sided<br>*Only common tests should be described solely by name; describe more complex techniques in the Methods section.*
☐	☒	A description of all covariates tested
☐	☒	A description of any assumptions or corrections, such as tests of normality and adjustment for multiple comparisons
☐	☒	A full description of the statistics including central tendency (e.g. means) or other basic estimates (e.g. regression coefficient) AND variation (e.g. standard deviation) or associated estimates of uncertainty (e.g. confidence intervals)
☐	☒	For null hypothesis testing, the test statistic (e.g. F, t, r) with confidence intervals, effect sizes, degrees of freedom and P value noted<br>*Give P values as exact values whenever suitable.*
☒	☐	For Bayesian analysis, information on the choice of priors and Markov chain Monte Carlo settings
☒	☐	For hierarchical and complex designs, identification of the appropriate level for tests and full reporting of outcomes
☐	☒	Estimates of effect sizes (e.g. Cohen's d, Pearson's r), indicating how they were calculated
☐	☒	Clearly defined error bars<br>*State explicitly what error bars represent (e.g. SD, SE, CI)*

Our web collection on statistics for biologists may be useful.

Software and code

Policy information about availability of computer code

Data collection	RNA seq and nanoString data were collected using the vendor's software Illumina HiSeq 2500 and nanoString nSolver version 3.
Data analysis	All statistical analyses concerning RNA-seq data were conducted in R version 3.3.3 using relevant Bioconductor packages (e.g. limma 3.30.13, edgeR 3.16.5). Co-expression networks were defined using the WGCNA 1.51 package in R. Pathway enrichment analysis was performed using CAMERA querying gene sets annotated in MSigDB v5.2. Protein interaction networks were generated using STRING version 10 (http://string-db.org/). Functional enrichment analysis to decipher functionally grouped gene ontology and biological process using ClueGO. GraphPad Prism Software 7, Cytoscape version 3.5.1 were also used.

For manuscripts utilizing custom algorithms or software that are central to the research but not yet described in published literature, software must be made available to editors/reviewers upon request. We strongly encourage code deposition in a community repository (e.g. GitHub). See the Nature Research guidelines for submitting code & software for further information.

Data

Policy information about availability of data

All manuscripts must include a data availability statement. This statement should provide the following information, where applicable:
- Accession codes, unique identifiers, or web links for publicly available datasets
- A list of figures that have associated raw data
- A description of any restrictions on data availability

The unprocessed transcriptomic data of this study have been deposited in the Gene Expression Omnibus under accession numbers GSE111006, GSE111010, GSE111016 and integrated in the series GSE111017. Other datasets analysed during the current study are available from the corresponding authors on reasonable request. Due to ethical concerns, supporting clinical data cannot be made openly available. The MEMOSA team can provide the data on request subject to appropriate approvals, after a formal application to the Oversight Group of the different cohorts through their respective corresponding author.

Field-specific reporting

Please select the best fit for your research. If you are not sure, read the appropriate sections before making your selection.

☒ Life sciences ☐ Behavioural & social sciences ☐ Ecological, evolutionary & environmental sciences

For a reference copy of the document with all sections, see nature.com/authors/policies/ReportingSummary-flat.pdf

Life sciences study design

All studies must disclose on these points even when the disclosure is negative.

Sample size	We estimated the sample size based on the paper Lee, M.-L. T., Whitmore, G. A. (2002). Power and sample size for DNA microarray studies, Statistics in Medicine, 21:3543-3570. We supposed sd=1 and mean = 1, E(R0) = 15 mean number of false positive , G0 = 15000 (number of undifferentially expressed genes in the experiment and the desired individual), power level is 0.80. The required sample size for each group was n = 18 and, hence, a total of 2n = 2(18) = 36 experimental units are required.
Data exclusions	40 samples were analyzed in the Singapore and Hertfordshire sarcopenia studies (SSS and HSS) and 39 samples in the Jamaica sarcopenia study (JSS). One sample from SSS was excluded from RNA sequencing analyses as it did not reach the expecting sequencing depth of 35 millions of uniquely mapped paired reads and only remaining samples with sufficient muscle biopsy material were analyzed in the SSS mitochondrial validation assays (expression, activity and NAD).
Replication	The RNAseq results from SSS were validated using Nanostring on the same samples. These results were further validated at a pathway level in two other collections of human muscle biopsies from HSS and JSS. Functional validation by measuring mitochondrial protein expression and mitochondrial complex activity was performed in the SSS.
Randomization	Given our experimental setting, groups were not randomized.
Blinding	People conducting experiments in the laboratories were blind to the sample labels

Reporting for specific materials, systems and methods

Materials & experimental systems

n/a	Involved in the study
☐	☒ Unique biological materials
☐	☒ Antibodies
☒	☐ Eukaryotic cell lines
☒	☐ Palaeontology
☐	☐ Animals and other organisms
☐	☒ Human research participants

Methods

n/a	Involved in the study
☒	☐ ChIP-seq
☒	☐ Flow cytometry
☒	☐ MRI-based neuroimaging

Unique biological materials

Policy information about availability of materials

Obtaining unique materials	Muscle biopsies were used for the different experiments and no tissue remained.

Antibodies

Antibodies used

Mouse monoclonal OXPHOS antibody cocktail (Abcam 110412), Rabbit polyclonal GAPDH antibody (Abcam 37168), Mouse monoclonal HSC70 antibody (clone B-6) (Santa Cruz Biotechnology 7298), Porin1 (Abcam #ab15895), citrate synthase (Abcam #ab96600), CD38 (R&D System #MAB24041)

Validation

- Mouse monoclonal OXPHOS antibody cocktail has been validated by the manufacturer in two dimension Blue Native PAGE analysis of fibroblasts that are normal and complex I deficient. Antibody profiles are available on Abcam website and it has been used for western blot applications in human samples for the following publications:
Newman LE et al. The ARL2 GTPase is required for mitochondrial morphology, motility, and maintenance of ATP levels. PLoS One 9:e99270 (2014).
Sánchez E et al. LYRM7/MZM1L is a UQCRFS1 chaperone involved in the last steps of mitochondrial Complex III assembly in human cells. Biochim Biophys Acta N/A:N/A (2012).

- Rabbit polyclonal GAPDH antibody (Abcam 37168) as been validated by the manufacturer by western blot Hela cells lysates. Antibody profiles are available on Abcam website. Antibody profiles are available on Abcam website and it has been used for western blot applications in human samples for the following publications:
Qiu M et al. JS-K promotes apoptosis by inducing ROS production in human prostate cancer cells. Oncol Lett 13:1137-1142 (2017).
Yuan Z et al. Overexpression of trefoil factor 3 (TFF3) contributes to the malignant progression in cervical cancer cells. Cancer Cell Int 17:7 (2017)
Wang Y et al. Downregulation of Mitochondrial Single Stranded DNA Binding Protein (SSBP1) Induces Mitochondrial Dysfunction and Increases the Radiosensitivity in Non-Small Cell Lung Cancer Cells. J Cancer 8:1400-1409 (2017)

- Mouse monoclonal HSC70 antibody (clone B-6) (Santa Cruz Biotechnology 7298) has been validated by the manufacturer by western blot in whole cell lysates. Antibody profiles are available on Abcam website and it has been used for western blot applications in human samples for the following publications:
Mazzoccoli, L. et al. 2018. MiR-29 silencing modulates the expression of target genes related to proliferation, apoptosis and methylation in Burkitt lymphoma cells. J. Cancer Res. Clin. Oncol.
Tian, Z. et al. 2018. Circulating ANGPTL2 Levels Increase in Humans and Mice Exhibiting Cardiac Dysfunction. Circ. J.. 82: 437-447.

Eukaryotic cell lines

Policy information about cell lines

Cell line source(s)

State the source of each cell line used.

Authentication

Describe the authentication procedures for each cell line used OR declare that none of the cell lines used were authenticated.

Mycoplasma contamination

Confirm that all cell lines tested negative for mycoplasma contamination OR describe the results of the testing for mycoplasma contamination OR declare that the cell lines were not tested for mycoplasma contamination.

Commonly misidentified lines
(See ICLAC register)

Name any commonly misidentified cell lines used in the study and provide a rationale for their use.

Palaeontology

Specimen provenance

Provide provenance information for specimens and describe permits that were obtained for the work (including the name of the issuing authority, the date of issue, and any identifying information).

Specimen deposition

Indicate where the specimens have been deposited to permit free access by other researchers.

Dating methods

If new dates are provided, describe how they were obtained (e.g. collection, storage, sample pretreatment and measurement), where they were obtained (i.e. lab name), the calibration program and the protocol for quality assurance OR state that no new dates are provided.

☐ Tick this box to confirm that the raw and calibrated dates are available in the paper or in Supplementary Information.

Animals and other organisms

Policy information about studies involving animals; ARRIVE guidelines recommended for reporting animal research

Laboratory animals

For laboratory animals, report species, strain, sex and age OR state that the study did not involve laboratory animals.

Wild animals

Provide details on animals observed in or captured in the field; report species, sex and age where possible. Describe how animals were caught and transported and what happened to captive animals after the study (if killed, explain why and describe method; if

released, say where and when) OR state that the study did not involve wild animals.

Field-collected samples

For laboratory work with field-collected samples, describe all relevant parameters such as housing, maintenance, temperature, photoperiod and end-of-experiment protocol OR state that the study did not involve samples collected from the field.

Human research participants

Policy information about studies involving human research participants

Population characteristics

Singapore Sarcopenia Study (SSS). Forty Chinese descent male aged 65-79 years with or without sarcopenia were recruited.
Hertfordshire Sarcopenia Study (HSS). Forty Caucasian male subjects aged 68-77 years with or without sarcopenia were included in the present study.
Jamaica Sarcopenia Study (JSS). Thirty-nine Afro-Caribbean male subjects with or without sarcopenia, 63-89 years old, were included in the present study.

Recruitment

Singapore Sarcopenia Study (SSS). Forty male subjects with and without a diagnosis of sarcopenia of comparable age group were recruited from two studies on healthy community-dwelling older men in Singapore (Singapore Sarcopenia Group and Aging in a Community Environment Study).
Hertfordshire Sarcopenia Study (HSS). One hundred and five healthy community dwelling older men, 68 -77 years old, who participated in the UK Hertfordshire Cohort Study were prospectively recruited into this present study (HSS) as previously described in detail (Patel et al., 2010).
Jamaica Sarcopenia Study (JSS). Male Afro-Caribbean participants with or without sarcopenia, were recruited through community-based (churches, community centers, senior citizen clubs) screening using the snowballing method for referrals.
